# Effectiveness and implementation of a primary healthcare intervention for multimorbidity and frailty among urban older adults in India: Protocol for the Multi-FrAME cluster randomized trial

**DOI:** 10.1371/journal.pone.0351110

**Published:** 2026-06-18

**Authors:** Jaya Singh Kshatri, Haimanti Bhattacharya, Siddhartha Goutam, Ashok Kumar Mahakuda, Rasmiranjan Nayak, Kavitha Adukkadukam Kambikanam, Tanveer Rehman, Abhinav Sinha, Mamata Manjari Sahu, Kshanaprava Mohakud, Purna Chandra Das, Sanghamitra Pati

**Affiliations:** 1 ICMR-Regional Medical Research Centre (Department of Health Research, Ministry of Health and Family Welfare, Government of India), Chandrasekharpur, Bhubaneswar, India; 2 Academy of Scientific and Innovative Research (AcSIR), Ghaziabad, Uttar Pradesh, India; 3 Maastricht University, The Netherlands; 4 IMS and SUM Hospital-II, Phulnakhra, Bhubaneswar, India; 5 Swami Vivekanand National Institute of Rehabilitation Training and Research (SVNIRTAR), Cuttack, India; 6 SUM Ultimate Medicare Hospital, Bhubaneswar, India; 7 Indian Council of Medical Research Centre, (Department of Health Research, Ministry of Health and Family Welfare, Government of India), New Delhi, India; University of Oxford, UNITED KINGDOM OF GREAT BRITAIN AND NORTHERN IRELAND

## Abstract

The worldwide impact of multimorbidity and frailty is accelerating among older adults, particularly in India, where healthcare systems face resource constraints. Multimorbidity defined as the coexistence of two or more chronic health conditions, and frailty—a state of heightened vulnerability—jointly result in functional disability, decreased quality of life, and increased healthcare use. Despite this, primary healthcare frameworks in India lack integrated geriatric care models. The study addresses this gap by evaluating a culturally appropriate, multicomponent intervention to improve quality of life related to health status and wellbeing among older adults in urban Odisha. The Multimorbidity-Frailty among elderly (Multi-FrAME) study is a phase 3, two-arm, cluster-randomized, observer-blinded trial that will be conducted across 10 urban Primary Healthcare Centres (UPHCs) in Bhubaneswar, Odisha. Eligible participants will be enrolled and randomized at the cluster level. The intervention group will receive a structured, monthly multicomponent package delivered through UPHCs whereas the control group will be given standard care. The primary outcome will include change in EuroQol 5-Dimension 5-Level (EQ-5D-5L) quality of life score at 6 and 12 months, and secondary outcomes will be a change in frailty, function, healthcare use, medication adherence and mortality. Data will be collected by trained, blinded assessors using validated tools and analyzed using mixed-effects regression models. Institutional ethical clearance was obtained for the study. The Multi-FrAME trial addresses a critical gap in geriatric care by evaluating a scalable, multicomponent intervention for frailty and multimorbidity within India’s primary healthcare system. Leveraging existing infrastructure and non-specialist staff, it integrates medical, nutritional, physical, and psychosocial support. Findings will inform policy and offer a replicable model for integrated elder care in Low and Middle Income Countries (LMICs). Trial Registration was done in Clinical Trial Registry of India (linked to WHO registry of trials) with registration no CTRI/2024/09/073487 on 5^th^ September,2024.

## Introduction

Globally people are ageing at an unprecedented rate, with the number of individuals aged 60 years and older expected to reach 2.1 billion worldwide by 2050. This demographic shift is particularly significant in Low and Middle Income Countries (LMICs), where health systems are often poorly equipped hence fail to address the complex needs of ageing populations. In India, the older adult population is about to rise from 103 million in 2020–319 million by 2050, representing nearly 20% of the total population [1].

Multimorbidity, defined as the co-occurrence of two or more chronic health conditions, is common among older adults. This is a long term health condition contributing to functional decline, polypharmacy, increased healthcare utilisation, and diminished quality of life [2,3]. Frailty on the other hand often coexist with multimorbidity to exacerbate adverse health outcomes. Frailty is a condition marked by decreased physiological reserve and increased vulnerability to stressors [4–6]. Evidence suggests a strong bidirectional relationship between multimorbidity and frailty, along with each condition accelerating the progression of the other [7,8].

The global estimation of frailty prevalence is 10% among community dwelling older adults with higher rates found among those with multimorbidity [5]. Frailty has been associated with lower scores in both objective and self-reported measures of health-related quality of life (HRQoL), particularly in LMICs where the availability of comprehensive geriatric care remains limited [7,9,10].

Multiple systematic reviews have highlighted the efficacy of multicomponent interventions—including exercise, nutritional supplementation, medication optimisation, and psychosocial support—in reducing frailty and improving functional status and HRQoL [11–15]. However, most of these evidences originate from high-income countries, raising questions about applicability and scalability in resource-constrained settings [12,16]. Therefore, context-specific interventions that are culturally appropriate, affordable, and system-integrated are urgently needed in LMICs.

In India, existing primary healthcare models are not adequately structured to manage complex age-related conditions like frailty and multimorbidity [6,17,18]. Despite the rollout of Health and Wellness Centres under the Ayushman Bharat programme, integrated care for older adults remains fragmented. Our previous work in rural Odisha “Assessment of Health Status of the Elderly in Tigiria block: a Syndemic approach” (AHSETS) study highlighted the high burden of multimorbidity and frailty, underscoring the urgent need for scalable, multidimensional interventions rooted in the primary care system [8,17,18].

The Multi-FrAME study addresses this evidence gap by testing the effectiveness of a multicomponent intervention delivered through urban primary healthcare centres (UPHCs) in Bhubaneswar, India. The intervention package includes components of medical management, nutritional support, physiotherapy, physical activity, and psychosocial counselling. The Multi-FrAME model aims to enhance quality of life and reduce the burden of frailty and multimorbidity in urban older adults by targeting both health system and patient-level determinants.

The primary objective includes to assess the effectiveness of the Multi-FrAME intervention in improving health-related quality of life, by measuring the EuroQol 5-Dimension 5-Level (EQ-5D-5L) index.

Secondary objectives include evaluating changes in frailty status, activities of daily living/instrumental activities of daily living (ADL/IADL), medication adherence, healthcare utilisation, expenditure, patient satisfaction, and all-cause mortality. Exploratory outcomes include changes in biochemical, haematological, and endocrine parameters that may mediate or reflect intervention effects.

## Materials and methods

### Trial design

The Multi-FrAME study is a phase 3, two-arm, cluster-randomized, parallel-group, observer-blinded trial. UPHCs in Bhubaneswar, the capital city of Odisha, India, serve as the unit of randomization. Clusters are randomized to either the intervention arm receiving the Multi-FrAME package or the control arm receiving standard care. The trial design aligns with the Consolidated Standards of Reporting Trials (CONSORT) extension for cluster trials and is reported in accordance with Standard Protocol Items: Recommendations for Intervention Trials (SPIRIT) 2013 guidelines (S1 File). The cluster design was chosen to minimize contamination, as the intervention involves PHC-level restructuring of care delivery. Outcome assessors will remain blinded to allocation, although participants and care providers will be unblinded due to the nature of the intervention.

### Study setting

The trial will be implemented across 10 selected UPHCs in Bhubaneswar.. These facilities, functioning as Health and Wellness Centers under India’s Ayushman Bharat program, serve urban catchment populations and represent a scalable platform for integrated geriatric care [18,19].

### Eligibility criteria

#### Inclusion criteria.

The study will include men and women aged 60 years or older who are residents of Bhubaneswar city and provide informed consent for participation [8]. The inclusion criteria also include participants diagnosed with multimorbidity (defined as having two or more chronic conditions) and having a Frailty Index score of ≥ 0.25 based on the deficit accumulation model. Multimorbidity will be assessed by the validated Multimorbidity Assessment Questionnaire for Primary Care (MAQ-PC) Plus tool [3].

#### Exclusion criteria.

Both male and female individuals will be excluded if they have severe cognitive impairment or dementia, as assessed by the Dementia Assessment by Rapid Test (DART). Individuals who are bedridden or have mobility-limiting conditions such as Grade III osteoarthritis, those with contraindications to physical activity, and those who are unable or unwilling to participate in follow-up assessments will also be excluded from the study [20].

### Intervention arm: Multi-FrAME package

Participants will receive a multicomponent intervention delivered by trained personnel through the UPHC, comprising the following key components:

#### Medical management.

Medical management intervention will include medication review, optimisation, adherence tracking via “health diary” which will be provided to them. The health diary would serve as a fidelity log and patient education tool. This will improve adherence to prescribed drugs and supplements and will enhance resilience among frail older adults with better functional outcomes compared to usual care without systematic supportive care [21,22].

#### Nutritional support.

The nutritional intervention includes dietary counselling and supplementation with Vitamin D3, calcium, iron, folic acid, and protein. This will provide individualized diet plans and targeted micronutrient and supplementation to address deficiencies and metabolic needs. By improving nutritional status, muscle strength and immunity will also improve along with capability of disease control with better functional and health outcome in frail elderly people [23–27].

#### Physiotherapy and physical activity.

The physical intervention program will include a standardized Timed Up-and-go (TUG) assessment to tailor home based exercise routines for frail older adults. Participants will receive individualized strength balance, endurance, stretching and cognitively engaging exercises matched to their functional capacity and comorbidity status. This structured, progressive approach will improve mobility, muscle strength, balance and mental agility while reducing fall risk and functional decline. [13,14,28].

#### Psychosocial support.

The psychosocial intervention integrates daily relaxation practices, need based psychological counselling and periodic group based social engagement to address emotional distress, stress, isolation and reduced coping capacity among the older people. Breathing, mediation and visualization techniques will enhance emotional regulation and reduce anxiety, while counselling supports adjustment to ageing and illness. Structured group activities, storytelling and therapeutic games will strengthen social connectedness and resilience. This intervention is expected to reduce psychological vulnerability, loneliness and functional decline leading to better quality of life [29–31].

The intervention package will be delivered after recruitment of the participants where a structured monthly contact schedule will be followed to maintain the health diary. A social media app based system for regular follow up will help to improve social networking and overall health monitoring among the frail older adults. The details of intervention delivery and frequency is summarised in (Table 1). All concomitant care is permitted during the trial.

**Table 1:** Components of the Multi-FrAME intervention.

Intervention Domain	Key Activities	Delivery Agent	Frequency	Mode of Delivery	Self –Care/ Caregiver Support
**Medical Management**	Medication review, adherence counselling, health diary	PHC Physician Study Nurse	Monthly	In-person	Self/Caregiver assisted
**Nutritional Support**	Dietary education, supplementation (Vitamin D3, Iron, Folic Acid, Calcium, Protein)	Dietitian Study team	Monthly	Oral counselling	Self
**Physiotherapy and Exercise**	Tailored resistance and aerobic exercises	Physiotherapist	Monthly + daily	In-person and home based	Self/Caregiver assisted
**Psychosocial Counselling**	Breathing/ mindfulness, storytelling, group sessions	Psychologist/ Social worker	Daily + Once in three months	In-person	Self
IEC* Tools and Health Diary	Health promotion content, adherence tracking, intervention logs	Study team	Continuous	Booklet, leaflet	Self

*IEC= Information, education and communication.

### Control arm: Routine standard care

Participants in the control arm will receive routine care provided through the UPHC. This typically includes episodic outpatient consultations, symptom-based prescribing, and referrals as required, without structured multidisciplinary input.

### Study outcomes

#### Primary outcome.

Primary outcome will include change in HRQoL that will be measured by the EQ-5D-5L index at baseline, 6 months, and 12 months [9,10].

EQ-5D-5L index score, will be analysed as a continuous variable. Mean differences between intervention and control groups at 6 and 12 months, as well as change from baseline, will be assessed using independent t-tests (or Mann–Whitney U tests if non-normal). A linear mixed-effects model will be used to estimate the treatment effect over time, accounting for repeated measures and baseline values. Results will be reported as adjusted mean differences with 95% confidence intervals and p-values.

#### Secondary outcomes.

Secondary outcome will include changes in frailty status and will be measured using a continuous Frailty Index based on the 40-item cumulative deficit model and assessing functional ability through the Lawton-Brody scale for ADL/IADL. Additional outcomes encompass healthcare utilisation metrics such as the number of outpatient consultations, emergency visits, and hospitalisations, alongside healthcare expenditure captured through direct costs borne by participants. Medication adherence will be assessed using the Medication Adherence Reporting Scale (MARS) and patient satisfaction will be measured through a validated satisfaction scale. Additionally, all-cause mortality will be monitored throughout the study to capture any intervention-related survival benefits [8].

Secondary outcomes will be analyzed according to their data type: continuous variables will be assessed using t-tests and linear mixed-effects models, categorical variables using chi-square tests and mixed-effects logistic regression, and ordinal outcomes using ordinal logistic models where appropriate. Treatment effects will be reported as adjusted mean differences or odds ratios with 95% confidence intervals, comparing intervention and control groups over time while adjusting for baseline differences.

#### Exploratory outcomes.

The exploratory outcomes of the Multi-FrAME trial focus on biological markers that may mediate or reflect the effects of the intervention. These include biochemical and haematological parameters such as hemoglobin, glycated hemoglobin (HbA1c), random blood sugar (RBS), lipid profile, and liver and kidney function tests. Micronutrient will be evaluated by measuring levels of vitamin D, iron, calcium, and magnesium levels [23–27]. In addition, hormone profile will include Thyroid Stimulating Hormone (TSH), Triiodothyronine (T3), Thyroxine (T4), and sex hormones. These outcomes are intended to provide mechanistic insights into how the multicomponent intervention impacts on physiological and metabolic health. A summary of outcome assessments with time points is provided in (Table 2).

**Table 2:** Outcome measures and time points.

Outcome Domain	Measure/ Tool	Assessment Time Points	Type
Health-related quality of life	EQ-5D-5L Index	Baseline, 6 months, 12 months	Primary
Frailty	Deficit Accumulation Frailty Index	Baseline, 6 months, 12 months	Secondary
Functional status	ADL/IADL (Lawton & Brody)	Baseline, 6 months, 12 months	Secondary
Healthcare utilisation	Custom questionnaire	Continuous	Secondary
Healthcare expenditure	Participant reports, receipts	Monthly	Secondary
Medication adherence	MARS Scale	Baseline, 6 months, 12 months	Secondary
Patient satisfaction	Satisfaction survey (Greenfield & Attkisson)	6 months, 12 months	Secondary
Mortality	Death registry and reports	Continuous	Secondary
Biochemical/haematological	Blood glucose, HbA1c, lipids, liver enzymes, kidney function, micronutrients	Baseline, 6 months, 12 months	Exploratory
Endocrine/hormonal	TSH, T3/T4, sex hormones.	Baseline, 6 months, 12 months	Exploratory

### Participant enrolment process and analysis plan

Participant enrolment, intervention provision and assessment will be done in three major time points: baseline (including screening, informed consent, frailty and multimorbidity assessment and baseline laboratory tests), six months (follow-up outcome assessments and laboratory tests) and twelve months (endline outcome and safety assessments).

The study will begin with a screening phase conducted through Urban Primary Health Centres (UPHCs) in Bhubaneswar. Permission will be obtained from the Medical Officer-in-Charge (MOIC) to screen older adults attending the Outpatient Department (OPD). In addition, ASHA workers will be engaged to mobilize older adults from their respective catchment areas, with support from community leaders and senior citizen clubs, who will be briefed about the study objectives and procedures.

Following screening, individuals who meet the eligibility criteria will be shortlisted. Eligible participants will be contacted telephonically and requested to visit the respective UPHCs for further study procedures and enrolment. In cases where telephonic contact is not feasible, ASHAs will facilitate communication and mobilization of eligible participants to the UPHCs.

**Fig 1:**
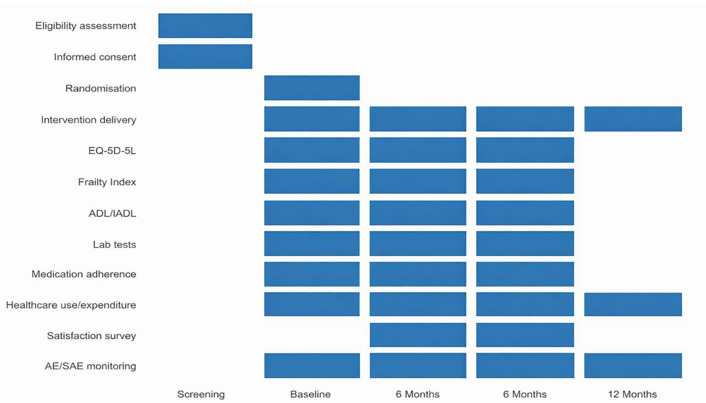
Standard Protocol Items: Recommendations for Interventional Trials (SPIRIT) schedule of enrolment, interventions and assessments.

The schedule of enrolment, interventions, and assessments is presented in (Fig 1).

**Fig 2:**
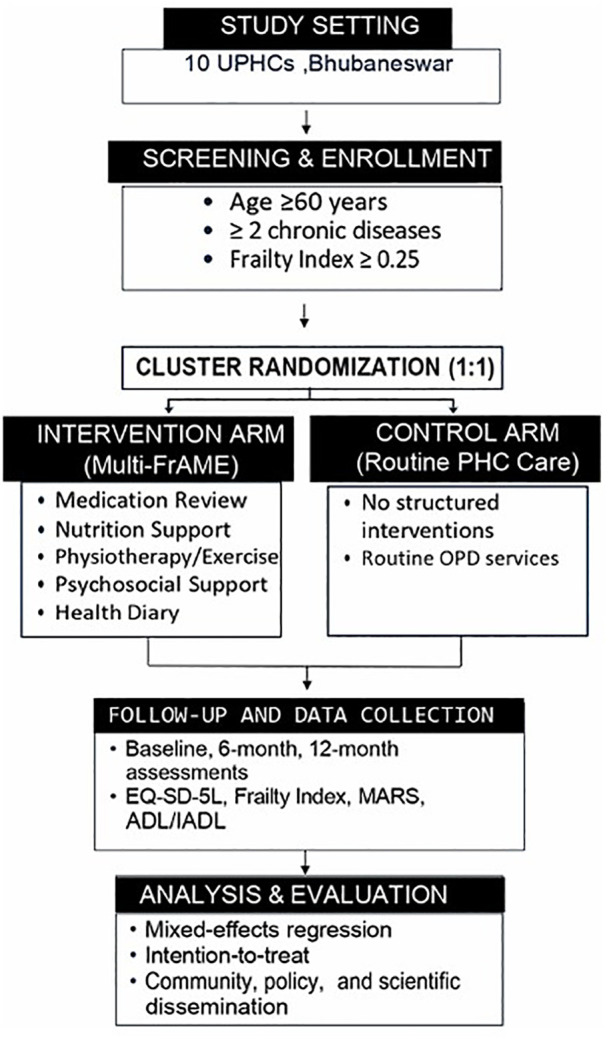
Multi-FrAME (Multimorbidity-Frailty among Elderly) study flow diagram.

A study flow diagram illustrating the study setting, screening and enrolment, cluster randomization, follow up assessment followed by mixed-effects regression analysis and dissemination is shown in (Fig 2).

An Intention to treat analysis will be performed. Detailed analysis plans for each outcomes and missing data handling are provided in the trial protocol as additional file.

Participant recruitment started from 15^th^ January, 2025 and will be completed by 30^th^ November 2025. Data collection will be completed by 30^th^ November, 2026 and results are expected by 31^st^ January, 2027.

#### Sample size.

The sample size calculation was done by detecting a clinically meaningful improvement in the primary outcome, the EQ-5D-5L health-related quality of life index. Assumptions used in the calculation: Mean EQ-5D-5L utility score in the control group: 0.87, SD = 0.17, minimum clinically important difference: 10% improvement, power: 90%, significance level (two-sided): 0.05, intra cluster correlation coefficient (ICC): 0.24 (31), expected attrition: 25%. Based on an a priori power calculation using a two-sided significance level of 0.05 and 90% power, the required sample size was estimated to be 430 participants, with 215 participants in each arm. This sample size was considered sufficient to detect a clinically meaningful difference in primary outcomes between the intervention and usual care groups.

These participants were distributed across 10 clusters, each comprising 43 individuals. Clusters were randomly allocated in a 1:1 ratio, with five clusters (n = 215 participants) assigned to the intervention arm and five clusters (n = 215 participants) to the control arm.

A two-sample t-test was first used to estimate the required sample size per arm assuming individual randomisation. This was then adjusted using the design effect to account for cluster randomisation: Design effect (DE)=1+(m − 1)×ICC (where *m* is the average cluster size). Final sample size estimates were inflated for the design effect and anticipated attrition.

### Assignment of interventions

#### Sequence generation and allocation.

Clusters (UPHCs) will be randomly assigned in a 1:1 ratio to either the intervention or control arm using computer-generated random numbers. Randomisation is stratified by UPHC characteristics (e.g., population served, catchment area demographics) to ensure balance across arms.

#### Allocation concealment.

An independent statistician who is not involved in implementation or data collection will conduct the randomisation. Allocation concealment will be maintained until clusters are enrolled.

#### Implementation.

The study coordinator will enrol PHCs and assign them to the intervention or control group according to the concealed allocation list.

#### Blinding.

Blinding of participants and providers is not feasible because of the nature of intervention. However, observer blinding will be maintained strictly. Outcome assessors responsible for administering the EQ-5D-5L, frailty index, and laboratory measurements will not be informed of group allocation.

Blinding will be further reinforced by segregated teams for intervention delivery and outcome assessment, lab sample coding and de-identified central analysis and health diaries collected in sealed envelopes to prevent unbinding during transport or entry.

Data analysts will also remain blinded to group allocation until the database is locked. Unblinding will be done if ethically required due to reporting of new health concerns.

### Data collection, management, and monitoring

#### Data collection methods.

Primary and secondary outcomes will be collected at 0, 6, and 12 months using validated instruments. Health diaries will be maintained by participant’s record adherence, intervention exposure, and healthcare events. Trained assessors, fluent in local languages will conduct structured interviews using standard operating procedures (SOPs) derived from the Manual of Operating Procedures (MOP). Refresher trainings and inter-rater reliability assessments will be held at every 6 months.

The study uses standardized measurements and questionnaires to assess participant characteristics, intervention fidelity, outcomes, and safety across the trial period. Eligibility is established at screening using an Eligibility CRF, while baseline socio-demographic characteristics, medical history, and health status will be collected using the Recruitment/Baseline CRF. Follow-up assessments will be conducted at 6 and 12 months using structured follow-up CRFs. All questionnaires will be administered by trained field investigators through interviewer led assessments to ensure consistency and minimize literacy-related bias.

The primary outcome, health-related-quality-of-life will be measured using the EQ-5D-5L,which is a standardized instrument capturing participant-reported health status across five dimensions using ordinal severity levels. Secondary outcomes include frailty, which will be assessed using a cumulative deficit Frailty Index based on 40 health-related variables, functional status measured using the Lawton and Brody ADL/IADL scale, medication adherence assessed with the Medication Adherence Reporting Scale using Likert-type responses, and patient satisfaction evaluated through structured interviews. Healthcare utilization and expenditure will be collected monthly using recall-based questionnaires and participant diaries, supplemented by healthcare records where available. Mortality and safety outcomes, including adverse and serious adverse events, will be monitored continuously, alongside standardized assessments of vital signs and laboratory investigations at baseline, 6, and 12 months.

#### Data management.

A secure and validated electronic data capture (EDC) platform will be used to enter data and unique IDs will be generated for anonymise participant data. Along with that a unified database management system (DBMS) with audit trails will ensure data integrity. Double-entry verification and logic checks will be used to identify inconsistencies.

Data will be stored securely for a minimum of five years after trial completion, in accordance with Indian Council of Medical Research (ICMR) and ethics committee requirements.

#### Monitoring and quality assurance.

Internal monitoring will be conducted monthly by the study QA team. External monitoring will be scheduled quarterly by an independent monitor. A Drug and Data Safety Monitoring Board (DSMB) meet will be conducted at predefined milestones (33%, 66%, 100%) to review safety and adherence data. Any protocol deviations will be logged and reviewed for their impact on study validity.

#### Ethical approval.

This study protocol has been approved by the Institutional Human Ethics Committee of the ICMR-Regional Medical Research Centre (ICMR-RMRC), Bhubaneswar (approval ID: ICMR-RMRC/IHEC-2024/015). All procedures adhere to the ICMR National Ethical Guidelines for Biomedical and Health Research Involving Human Participants (2017), and conform to the principles of the Declaration of Helsinki.

#### Informed consent.

Written informed consent will be collected from all participants before any trial-related procedures. Field investigators, trained in ethics and communication will explain the study details in participants’ preferred language (Odia, Hindi, or English), including consent for biological samples and testing, and storage. Visual aids and lay explanations will be used to enhance comprehension. All participants will be provided the copies of signed consent form and participant information sheet.

In cases of limited literacy, a witness is present, and thumbprints are accepted per regulatory standards.

#### Confidentiality and data protection.

Participant confidentiality will be protected at all stages of the study. All identifiable information will be stored separately from analytic datasets and accessed only by authorised personnel. Databases will be password-protected, encrypted, and backed up regularly. All physical records will be stored in locked cabinets at the central coordinating centre. Results will be reported in aggregate form without disclosing individual identities. Data will be retained for at least five years post-trial, as per ICMR guidelines.

#### Dissemination plan.

Findings from this trial will be disseminated through publications in international peer-reviewed journals and presentations at national and international scientific conferences. Policy briefs will be developed, and results will be shared during stakeholder meetings with state and national health authorities. Additionally, a lay summary of the findings will be provided to participants and community representatives.

### Data monitoring and auditing

#### Data and safety monitoring board (DSMB).

An independent DSMB will supervise safety, adherence, and interim efficacy data during 33% enrolment, 66% enrolment and at trial completion. The board comprises senior clinicians, statisticians, and ethicists. The DSMB is empowered to recommend continuation, modification, or early termination of the trial based on predefined stopping rules (e.g., safety signals, futility).

#### Auditing and monitoring.

This include internal and external monitoring. During internal monitoring monthly site-level checks will be done by the QA team. External monitoring will be conducted by trained monitors unaffiliated with the trial team, focusing on consent documentation, protocol adherence, and CRF accuracy. All adverse events, protocol deviations, and data inconsistencies will be reviewed by the core coordinating team and rectified promptly.

### Harms and adverse events

#### Definitions.

Any undesirable medical occurrence during the trial, regardless of causal relationship is defined as Adverse Event (AE) whereas any event that leads to death, life-threatening, needs hospitalisation, or causes significant disability is termed as Serious Adverse Event (SAE).

#### Reporting procedures.

Reporting for any AEs and SAEs will be done within 24 hours to the Principal Investigator and documented in the Case Report Forms (CRFs). SAEs will also be reported to the Ethics Committee within seven working days. Causality will be assessed by a blinded clinical adjudication panel. All SAEs are followed to resolution or stabilisation.

#### Anticipated harms.

Risks associated with the intervention include supplement-related effects: (e.g., hypercalcemia, GI disturbances from iron or calcium), exercise-related injuries: e.g., musculoskeletal strain, fatigue), Emotional distress (arising from counselling sessions).

#### Mitigation strategy.

Mitigation strategy consist of individualised dosing based on lab values, pre-exercise screening and physiotherapist supervision and psychological first-aid protocols and escalation pathways. The trial will be conducted at public health facilities and all ancillary care will be provided there, free of cost to the participants.

## Discussion

India, like many LMICs, is experiencing a demographic transition with a rapidly growing older adult population. This shift is accompanied by an increased burden of multimorbidity and frailty conditions that co-occur, interact, and complicate health management in older individuals [2,5,6,8]. Despite this growing challenge, primary healthcare systems in India remain largely oriented toward acute episodic care and single-disease frameworks [7,17,18]. The Multi-FrAME trial is designed to address this gap by evaluating a pragmatic, multicomponent intervention for the control and management of multimorbidity and frailty among older adults through urban primary healthcare centres. Though high-income countries have demonstrated the evidences of effectiveness of multidimensional interventions involving physical activity, medication review, nutritional support, and psychosocial interventions in improving frailty and quality of life [11,15,29,30], the LMIC-specific evidence is limited, and there are few rigorously designed trials that test such interventions within routine primary care systems. Existing studies in India have primarily been observational or lacked control groups, and many have focused on single components such as physical function or nutrition [8,17–19]. Multi-FrAME builds on this foundation, integrating lessons from our previous work in Odisha (e.g., the AHSETS study) and tailoring delivery models to the Indian public health context [8,17,18].

This protocol describes a cluster-randomised, phase 3 trial that influences existing UPHC infrastructure and includes several novel elements. First, the intervention is holistic and tailored, addressing medical, functional, nutritional, and psychosocial domains. Second, the implementation design emphasises scalability where intervention components are delivered by trained non-specialist staff and supported with simple tools such as health diaries [21,22]. Third, the study includes a comprehensive set of outcomes ranging from patient-reported quality of life to biochemical parameters to provide both effectiveness and mechanistic insights.

Several features enhance the scientific rigour of the trial. Randomisation at the UPHC (cluster) level reduces contamination between intervention and control groups, and observer blinding of outcome assessors minimises measurement bias. The sample size is adequately powered, accounting for intra-cluster correlation and expected attrition. Additionally, fidelity mechanisms such as logs, monthly reviews, and monitoring visits have been built into the delivery strategy.

Nonetheless, certain limitations warrant consideration. As with most complex interventions, blinding of participants and providers is not feasible, which may introduce performance bias. To mitigate this, outcomes are collected by independent assessors using validated tools [8,10,20]. Additionally, although the intervention is embedded in UPHCs, sustainability and scalability beyond the trial setting will require engagement with policy-makers and potential system-level reforms. We also recognise that the trial is limited to urban PHCs in a single city; findings may need contextual adaptation for rural or peri-urban populations [19].

The COVID-19 pandemic has accelerated awareness of older adult vulnerabilities and the importance of decentralised chronic disease care. The Multi-FrAME model aligns with India’s evolving public health priorities under the Ayushman Bharat initiative and offers a framework for integrated geriatric care at the primary care level [19].

Once completed, this trial is expected to produce robust evidence on the effectiveness, feasibility, and safety of a scalable frailty-multimorbidity management package. It will also help in guiding the development of future models of community-based geriatric care in LMICs and contribute to global implementation science on ageing.

## Supporting information

S1 File Spirit checklist. (PDF)

S2 File Protocol. (PDF)
