# Supplementary material for: Effectiveness and implementation of a primary healthcare intervention for multimorbidity and frailty among urban older adults in India: Protocol for the Multi-FrAME cluster randomized trial
Source: PLoS One. 2026 Jun 18;21(6):e0351110. doi: 10.1371/journal.pone.0351110 (PMC13278581; doi:10.1371/journal.pone.0351110)
Supplement: S2 File — (PDF) [file pone.0351110.s002.pdf]

TRIAL PROTOCOL:

EFFECTIVENESS AND IMPLEMENTATION OF A PRIMARY HEALTHCARE INTERVENTION FOR SCREENING AND MANAGEMENT OF MULTIMORBIDITY AND FRAILTY AMONG URBAN OLDER ADULTS OF BHUBANESWAR: THE MULTI-FRAME STUDY

CONFIDENTIAL

Version 3.5

Version date: 18.06.2025

INVESTIGATORS DETAILS

Name	Role	Qualifications	Designation
Dr. Jaya Singh Kshatri	Principal Investigator	MBBS, MD (Community Medicine), PG Diploma (Geriatric Medicine)	Scientist-C (Med), ICMR-RMRC Bhubaneswar
Dr Susanta Kumar Swain	Co-Investigator	MBBS, Master in Advanced Epidemiology	Additional Director to Health Services, Government of Odisha, Bhubaneswar.
Dr. Tanveer Rehman	Co-investigator	MBBS, MD (PSM)	Scientist-B, ICMR-RMRC, Bhubaneswar
Dr. Purna Chandra Dash	Co-investigator	MBBS, MD	Consultant Geriatrician, IMS&SUM Hospital, Bhubaneswar
Dr. Siddharth Gautam	Co-investigator	MBBS, MD (Pharmacology)	Associate Professor, IMS&SUM Hospital, Cuttack
Dr. Mamata Manjari Sahu	Co-investigator	BPT, MPT, PhD (Ortho PT)	Senior Physiotherapist, SVNIRTAR, Cuttack
Dr. Kshanaprava Mohakud	Co-investigator	MOT(Rehabilitation) PhD	Occupational Therapist, SVNIRTAR, Cuttack

CONFIDENTIAL

INVESTIGATOR’S SIGNATURE

[add digital signature]

TABLE OF CONTENTS

1 PROTOCOL SUMMARY 8
1.1 Synopsis 8
1.2 Schema 9
1.3 Schedule of Activities 10
2 INTRODUCTION 11
2.1 Study Rationale 11
2.2 Background 12
2.3 Risk Benefit Assessment 15
3 OBJECTIVES AND ENDPOINTS 21
3.1 Primary Objectives 22
3.2 Secondary Objectives 22
3.3 Biochemical and Haematological Outcomes 23
4 STUDY DESIGN 24
4.1 Overall Design 24
4.2 Scientific Rationale for Study Design 25
4.3 Justification for Intervention 25
4.4 End-of-Study Definition 26
5 STUDY POPULATION 26
5.1 Inclusion Criteria 26
5.2 Exclusion Criteria 26
5.3 Lifestyle Considerations 27
5.4 Screen Failures 27
5.5 Strategies for Recruitment and Retention 28

CONFIDENTIAL

6 STUDY INTERVENTION(S) OR EXPERIMENTAL MANIPULATION(S) 28
6.1 Study Intervention Overview 28
6.2 Administration and/or Dosing 29
6.3 Fidelity 30
6.4 Measures to Minimize Bias 31
6.5 Study Intervention/Experimental Manipulation Adherence 32
7 Concomitant Therapy 32
8 DISCONTINUATION AND PARTICIPANT DISCONTINUATION/WITHDRAWAL 32
8.1 Discontinuation of Study Intervention/Experimental Manipulation 32
8.2 Participant Discontinuation/Withdrawal from the Study: 32
8.3 Procedures for Discontinuation/Withdrawal 33
9 STUDY ASSESSMENTS AND PROCEDURES 33
9.1 Endpoint and Other Non-Safety Assessments 33
9.2 Safety Assessments 35
9.3 Data Collection and Management 36
10 Adverse Events and Serious Adverse Events 37
10.1 Definition of Adverse Events (AEs) 37
10.2 Definition of Serious Adverse Events (SAEs) 37
10.3 Classification of an Adverse Event 37
10.4 Time Period and Frequency for Event Assessment and Follow-Up 38
10.5 Adverse Event Reporting 38
10.6 Serious Adverse Event Reporting 39
10.7 Reporting Events to Participants 39
10.8 Events of Special Interest 39
11 Unanticipated Problems 40
11.1 Definition of Unanticipated Problems 40

CONFIDENTIAL

11.2 Unanticipated Problems Reporting 40
11.3 Reporting Unanticipated Problems to Participants 40
12 STATISTICAL CONSIDERATIONS 40
12.1 Statistical Hypotheses 40
12.2 Sample Size Determination 41
12.3 Populations for Analyses 43
12.4 Statistical Analyses 43
13 SUPPORTING DOCUMENTATION AND OPERATIONAL CONSIDERATIONS 48
13.1 Regulatory, Ethical, and Study Oversight Considerations 48
13.2 Informed Consent Process 49
13.3 Study Discontinuation and Closure 49
13.4 Confidentiality and Privacy 49
13.5 Future Use of Stored Specimens and Data 50
13.6 Key Roles and Study Governance 50
13.7 Safety Oversight 51
13.8 Clinical Monitoring 51
13.9 Quality Assurance and Quality Control 52
13.10 Data QA/QC: 52
13.11 Data Handling and Record Keeping 52
13.12 Protocol Deviations 52
13.13 Publication and Data Sharing Policy: 53
13.14 Conflict of Interest Policy: 53
14 Additional Considerations 53
14.1 Cultural Sensitivity: 53
14.2 Participant Burden 53

CONFIDENTIAL

14.3 Contingency Planning: 53
14.4 Environmental Considerations 54
14.5 Technology and Innovation 54
15 Abbreviations and Special Terms 54
16 REFERENCES 57
17 ANNEXURES 60

CONFIDENTIAL

1 PROTOCOL SUMMARY

1.1 SYNOPSIS

Title: Effectiveness and Implementation of a Primary Healthcare Intervention for Screening and Management of Multimorbidity and Frailty among Urban Older Adults: The Multi-FrAME Study

Design: Phase 3 cluster randomized, parallel arm, observer-blinded trial.

Objective: To assess the effectiveness of the Multi-FrAME intervention package in improving health outcomes for older adults with multimorbidity and frailty.

Setting: Urban Primary Healthcare Centres (PHCs) in Bhubaneswar, India.

Participants: Older adults (aged 60+) with multimorbidity and frailty (Frailty Index ≥ 25), without dementia, and not bedridden.

Intervention: The Multi-FrAME package, which includes medical management, nutritional support, physiotherapy, physical activity, and psychosocial counselling.

Comparator: Standard routine care.

Primary Outcome: Improvement in health-related quality of life, measured by EQ-5D-5L index scores.

Secondary Outcomes:

- Changes in frailty index scores
- ADL/IADL scores
- Healthcare utilization and expenditure
- Patient satisfaction
- Medication adherence
- Mortality rates

Data Collection: Baseline, 6, and 12 months.

Safety Monitoring: Regular assessment of adverse events and serious adverse events throughout the study.

Analysis: Intention-to-treat analysis, with planned interim analysis after 50% completion of the 12-month follow-up.

Ethical Considerations: Approved by the Institutional Human Ethical Committee of ICMR-RMRC Bhubaneswar, adhering to ICMR guidelines [**ICMR-RMRC/IHEC-2024/015**].

CONFIDENTIAL

This trial aims to provide insights into effective management strategies for older adults with complex health needs in a primary care setting.

CTRI Registration: CTRI/2024/09/073487

1.2 SCHEMA

i. **Enrollment:**
   a. Screening for eligibility using the MAQ-PC plus tool, frailty index and DART test.

ii. **Randomization:**
   a. Clusters (PHCs in Bhubaneswar) randomly assigned to either:
      i. **Intervention Group:** Multi-FrAME package
      ii. **Control Group:** Standard routine care

iii. **Intervention Components (Multi-FrAME):**
   a. Medical management
   b. Nutritional support
   c. Physiotherapy and physical activity
   d. Psychosocial counseling

iv. **Data Collection Time Points:**
   a. Baseline
   b. Follow-up at 6 months
   c. Follow-up at 12 months

v. **Outcome Measures:**
   a. **Primary:** EQ-5D-5L index scores
   b. **Secondary:** Frailty index, ADL/IADL scores, healthcare utilization/expenditure, patient satisfaction, medication adherence, mortality

vi. **Monitoring and Safety:**
   a. Monthly contact sessions for intervention fidelity and data collection
   b. Continuous monitoring of AEs and SAEs
   c. Training of all sites on identifying AE/SAE
   d. Overview by DSMB

vii. **Analysis:**
   a. Intention-to-treat approach

CONFIDENTIAL

b. Interim analysis after 50% completion of the 12-month follow-up

viii. **Completion:**

a. Endline assessments at 12 months

b. Data analysis and dissemination of findings

1.3 Schedule of Activities

Activity	*Screening*	*0 Month*	*6 Months*	*12 Months*
Eligibility Assessment	✓			
Informed Consent	✓			
Randomization		✓		
Medical Assessment		✓	✓	✓
EQ-5D-5L Quality of Life Assessment		✓	✓	✓
Frailty Index Evaluation		✓	✓	✓
ADL/IADL Assessment		✓	✓	✓
Healthcare Utilization & Expenditure		✓	✓	✓
Medication Adherence Monitoring		✓	✓	✓
Patient Satisfaction Survey			✓	✓
Intervention Delivery (Multi-FrAME)		✓	Continuous	Continuous
Adverse Event Monitoring		Continuous	Continuous	Continuous
Lab Tests & Biomarker Collection		✓	✓	✓

Interim Analysis	After 50% of 12-months
Final Data Collection & Analysis	✓

2 INTRODUCTION

2.1 Study Rationale:

The increasing number of older adults in low- and middle-income countries (LMICs) like India will impose a significant burden on health systems in the future. Complex aging pathways, including multimorbidity, frailty, and other syndromes, contribute to this burden. In India alone, the population aged 60 and above is projected to rise dramatically from 103 million to 319 million by 2050, representing nearly 20% of the total population (United Nations, 2022).

Multimorbidity, defined as the coexistence of two or more chronic conditions, and frailty, characterized by increased vulnerability to stressors due to diminished physiological reserve, are prevalent worldwide. Studies reveal that over 70% of older adults in high-income countries experience multimorbidity, with significant prevalence also observed in LMICs (Barnett et al., 2012; Marengoni et al., 2011).

CONFIDENTIAL

Frailty is particularly relevant in individuals with multimorbidity, as it often exacerbates health outcomes, including increased risk of hospitalization, disability, and mortality. Identifying and managing frailty in this group is crucial as a public health strategy. Research shows a strong association between multimorbidity and frailty, where frailty significantly worsens the impact of chronic diseases (Fried et al., 2001).

Frailty adversely affects numerous health outcomes, notably reducing patient-reported quality of life (QoL). Frail individuals report lower scores in mental and emotional well-being, impacting their overall life satisfaction (Collard et al., 2012). This highlights the need for interventions that address frailty to improve QoL and health outcomes.

Systematic reviews indicate various intervention types, such as exercise programs, nutrition support, and comprehensive geriatric assessments, can mitigate frailty and enhance QoL and other

patient-reported outcome measures (PROMs). However, the evidence predominantly stems from high-income countries, leaving a gap regarding LMICs (Clegg et al., 2013; Puts et al., 2017).

Further trials are essential, particularly focusing on frailty-related interventions in LMICs where evidence is scarce. In India, there is a dearth of research exploring these interventions. Systematic reviews underscore the urgent need for studies from this region to contribute valuable data (Abizanda et al., 2016; Apóstolo et al., 2018).

This trial intends to address this gap by evaluating a comprehensive intervention targeting multimorbidity and frailty among older adults in urban India. The findings aim to inform policy and practice, ultimately improving healthcare delivery and outcomes for this vulnerable population.

2.2 BACKGROUND:

The increasing prevalence of age-related syndromes, such as multimorbidity and frailty, presents significant challenges to global healthcare systems, particularly in low- and middle-income countries (LMICs) like India. As the demographic shifts towards an older population, with projections showing the number of adults aged 60 and above rising to 319 million by 2050 (United Nations, 2022), addressing the complex needs of older adults becomes crucial.

Multimorbidity refers to the coexistence of two or more chronic medical conditions within one individual, often leading to complex healthcare needs and management challenges. Globally, more than 70% of older adults experience multimorbidity, which is associated with a higher risk of adverse outcomes including increased healthcare utilization, polypharmacy, and reduced quality of life (Barnett et al., 2012). In India, a significant proportion of the elderly population is affected, highlighting the urgent need for integrated care approaches (Pramesh et al., 2014).

Frailty, often coexisting with multimorbidity, is characterized by decreased physiological reserve and increased vulnerability to stressors, leading to adverse health outcomes such as falls, disability, hospitalization, and mortality (Fried et al., 2001). Frailty affects approximately 10% of community-dwelling older adults globally (Collard et al., 2012), and its prevalence is even higher among those with multiple chronic conditions (Chang et al., 2019).

Importantly, frailty profoundly impacts patient-reported outcomes, significantly diminishing quality of life across physical, mental, and social domains (Dent et al., 2016). Frail individuals often

experience lower satisfaction levels and poorer mental health outcomes, underscoring the importance of targeted interventions.

Several intervention strategies have demonstrated potential in mitigating frailty and its effects. Comprehensive geriatric assessments, multidisciplinary care models, and personalized interventions focusing on exercise, nutrition, and social support have shown promise in improving health outcomes for frail older adults (Clegg et al., 2013; Puts et al., 2017). However, most evidence is derived from high-income countries, leaving a substantial gap regarding applicability in LMICs (Hoogendijk et al., 2019).

Gaps in Current Evidence

Systematic reviews consistently point to a lack of research on frailty-related interventions in LMICs. The unique cultural, economic, and healthcare infrastructures in these regions necessitate context-specific studies to develop sustainable and effective models of care (Abizanda et al., 2016; Apóstolo et al., 2018). In India, where health systems already face numerous challenges, research into culturally appropriate and scalable interventions remains crucial. This trial aims to fill this gap by implementing and evaluating a comprehensive intervention for frail older adults with multimorbidity, contributing valuable insights to the global evidence base.

The management of frailty in older adults is multifaceted, involving various intervention strategies that address medical, physical, nutritional, and psychosocial aspects. Evidence from textbooks and systematic reviews highlights the effectiveness of different approaches, often recommending a combination for optimal outcomes.

Medical Management- Effective management of existing chronic conditions and medication adherence are crucial in preventing the progression of frailty. Ensuring regular review and optimization of medication regimens can reduce polypharmacy risks, improve health outcomes, and enhance quality of life (Maher et al., 2014). Systematic reviews suggest that medication management, including deprescribing unnecessary medications, significantly impacts frailty levels (Gnjidic et al., 2012).

Physiotherapy and Physical Activity/Exercise- Exercise and physiotherapy are fundamental components in managing frailty, with substantial evidence supporting their role in improving muscle strength, balance, and overall physical function. Resistance and aerobic exercises, tailored to

CONFIDENTIAL

individual capabilities, have been shown to reduce frailty and improve independence in daily activities (de Labra et al., 2015). Comprehensive exercise programs are recommended in geriatric care models (Landi et al., 2018).

Nutritional Supplementation- Nutritional interventions, including supplementation with Vitamin D3, iron, folic acid, calcium, and protein, play a vital role in managing frailty. Vitamin D3 supplementation has been linked to improved muscle function and reduced fall risk (Bischoff-Ferrari et al., 2016). Iron and folic acid can address anemia-related fatigue, enhancing energy and activity levels (Fairweather-Tait et al., 2014). Calcium and protein intake are essential for maintaining bone health and muscle mass, crucial in slowing frailty progression (Bauer et al., 2013).

Psycho-Social Counselling Based Interventions- Psycho-social interventions are critical in addressing the mental and emotional aspects of frailty. Counseling and support groups improve social connectivity, reduce depression, and enhance coping mechanisms, all contributing to better quality of life (Wuthrich et al., 2015). Cognitive-behavioral therapy has shown benefits in reducing anxiety and improving self-efficacy among older adults (Chodosh et al., 2015).

Despite the available evidence, most studies are from high-income countries, necessitating further trials in LMICs like India. Limited context-specific research exists for these interventions, highlighting an urgent need for studies exploring their applicability in diverse settings (Apóstolo et al., 2018; Hoogendijk et al., 2019).

This trial aims to fill this gap by implementing and evaluating a comprehensive intervention for frail older adults with multimorbidity in an urban Indian setting, providing critical insights for global health policy.

AHSETS Study: Our previous work exploring the complex interactions between aging syndromes, including multimorbidity and frailty, within the AHSETS community-based cohort, underscores the need for comprehensive primary care interventions targeting multiple conditions and outcomes. These interventions should transition from disease-specific approaches to patient-centered models that emphasize early identification, integrated care, and coordination among multidisciplinary teams (Kshatri et al., 2020; Kshatri et al., 2021). Tailored care can significantly improve health outcomes, reduce costs, and enhance quality of life for older adults (Kshatri et al., 2020).

CONFIDENTIAL

Urban primary healthcare services, especially health and wellness centers, are crucial in delivering accessible care to older adults. However, there is limited evidence on the effectiveness of managing multimorbidity and frailty within these settings. Current primary care models often lack the comprehensive, integrated care required by these patients (Kshatri et al., 2023).

This clinical trial protocol aims to address this gap by developing and evaluating an integrated, multidimensional intervention for older adults with multimorbidity and frailty in urban primary healthcare centers. The focus is on delivering proactive, coordinated care through a patient-centered approach involving multidisciplinary teams, thereby improving management and reducing the burden on healthcare systems in LMICs like India.

2.3 Risk Benefit Assessment

2.3.1 Known Potential Risks:

A. Nutritional Supplementation

i. **Vitamin D3 (Cholecalciferol)**

- **Hypercalcemia:** Excessive intake can lead to elevated calcium levels, causing nausea, vomiting, weakness, frequent urination, and kidney damage. Symptoms may appear when serum calcium exceeds 10.5 mg/dL (Jones, 2008).
- **Hypervitaminosis D:** Chronic high doses can result in toxicity, symptoms include confusion, disorientation, and cardiovascular complications (Holick, 2007).
- **Gastrointestinal Disturbances:** Constipation, diarrhea, or abdominal discomfort may occur (Vieth, 1999).

ii. **Calcium**

- **Kidney Stones:** Increased risk of calcium oxalate stones, leading to renal colic (Curhan et al., 1997).

CONFIDENTIAL

- **Hypercalcemia:** Similar symptoms to Vitamin D3 excess.
- **Gastrointestinal Symptoms:** Bloating, gas, or constipation, especially at high doses (Straub, 2007).

iii. **Iron**

- **Gastrointestinal Upset:** Nausea, vomiting, diarrhea, and constipation (Haas & Brownlie, 2001).
- **Iron Overload:** Conditions like hemochromatosis can occur with chronic excessive intake, leading to organ damage (Borgna-Pignatti et al., 2010).
- **Staining of Teeth:** Liquid supplements may cause staining (Provan, 1999).

iv. **Folic Acid**

- **Masking of Vitamin B12 Deficiency:** Can lead to neurological damage if B12 deficiency goes undiagnosed (Chanarin & Metz, 1997).
- **Gastrointestinal Symptoms:** Mild bloating or gas may occur (Tamura & Picciano, 2006).

B. Physical Exercise/Physiotherapy Risks

- **Musculoskeletal Injuries:** Risks of strains, sprains, especially in those unaccustomed to physical activity (Macera et al., 2003).
- **Cardiovascular Events:** Exercise can trigger events like myocardial infarction in at-risk populations (Thompson et al., 2007).
- **Fatigue and Overexertion:** Inadequate pacing may lead to dizziness or syncope (O'Halloran et al., 2007).

C. Psychosocial Support Risks

- **Emotional Distress:** Discussions may evoke anxiety or depression, especially if participants struggle with conditions (Baikie & Wilhelm, 2005).

CONFIDENTIAL

- **Non-compliance with Intervention:** Perceived inadequacy may lead to withdrawal (Tong et al., 2012).

- **Dependence on Support Services:** Participants might rely excessively on services, hindering independence (Cumming, 1996).

D. General Risks

- **Withdrawal from Trial:** Adverse events or perceived ineffectiveness may lead to withdrawal.

- **Infections:** Risk of infections with interventions involving close contact, particularly for immunocompromised individuals (Geraci et al., 2009).

- **Psychological Effects of Monitoring:** Frequent evaluations could cause health anxiety (Clarke et al., 2013).

2.3.2 Known Potential Benefits:

Systematic reviews suggest that interventions targeting frailty and multimorbidity significantly improve quality of life and decrease mortality and morbidity rates. Although most evidence originates from high-income countries, the insights can guide interventions globally, including in India.

i. **Improvement in Frailty:**

- Comprehensive interventions can reduce frailty and improve physical function in older adults. Studies show enhanced muscle strength and mobility (Clegg et al., 2013; Puts et al., 2017).

ii. **Enhanced Quality of Life:**

- Targeted interventions lead to improved mental and emotional well-being. Frail individuals often experience better life satisfaction and reduced depression (Dent et al., 2016).

iii. **Reduction in Morbidity and Mortality:**

CONFIDENTIAL

- Addressing multimorbidity can decrease hospitalization rates and overall mortality. Integrated care models improve health outcomes by managing chronic conditions effectively (Fried et al., 2001; Adlins et al., 2014).

iv. Local Relevance:

- This trial addresses the critical gap in evidence for interventions tailored for older adults in India, contributing valuable data to the field and informing policy development (Kshatri et al., 2020).

v. Improved Healthcare Utilization:

- Coordinated care reduces unnecessary healthcare use and costs, optimizing resource allocation for better overall efficiency (Hoogendijk et al., 2019).

2.3.3 ASSESSMENT OF POTENTIAL RISKS AND BENEFITS

The assessment of potential risks and benefits will be an ongoing process throughout the trial to ensure participant safety and the efficacy of the intervention. A multidisciplinary team including clinical trial coordinators, physicians, physiotherapists, dietitians, and social workers will conduct this assessment.

A. Roles and Responsibilities

a) **Clinical Trial Coordinator/Manager:**

- Oversee the risk/benefit assessment process.
- Ensure adherence to ethical guidelines and regulatory requirements.
- Maintain communication with the Institutional Ethical Committee (IEC) and other regulatory bodies.

b) **Principal Investigator (PI):**

- Lead the overall assessment and make final decisions regarding participant safety.

CONFIDENTIAL

- Review data from adverse event reports and assess the need for protocol modifications.

c) **Medical Staff (Physicians, Nurses):**

- Conduct initial health screenings and ongoing health assessments of participants.
- Monitor and document any adverse events.

d) **Physiotherapists:**

- Evaluate physical capabilities and risks associated with exercise regimens.
- Monitor participants during physical activities to minimize injury risks.

e) **Dietitians:**

- Assess nutritional needs and risks associated with supplementation.
- Provide education on dietary modifications.

f) **Social Workers:**

- Evaluate psychosocial factors impacting participant adherence and well-being.
- Provide support and resources to address emotional distress related to chronic conditions.

B. Stages of Assessment

i. **Pre-Enrollment Stage:**

a. Conduct comprehensive assessments of each participant's health status, medical history, current medications, and psychosocial factors.

b. Utilize standardized assessment tools (e.g., frailty index, quality of life scales) to establish baseline data.

CONFIDENTIAL

ii. **Initial Assessment (During Enrollment):**

a. Review participant eligibility and identify contraindications for specific intervention components.

b. Document pre-existing conditions that may increase risk.

iii. **Ongoing Monitoring (Throughout the Trial):**

a. Regular health check-ups scheduled at 6, and 12 months to track health changes, adherence, and adverse events.

b. Continuous documentation and reporting of any adverse effects to the PI and trial coordinator.

c. Continuous overview and monitoring by an independent Drug, Data Safety and Monitoring Board (DSMB)

iv. **Post-Assessment Reviews (After Each Assessment Phase):**

a. Conduct review meetings involving the multidisciplinary team to evaluate data from health assessments, adverse event reports, and participant feedback.

b. Discuss trends, potential safety issues, and the effectiveness of the intervention.

C. Actions Following Assessment

i. **Intervention Adjustments:**

a. Implement modifications if significant risks are identified (e.g., adjusting dosages, altering exercise regimens).

b. Protocol amendments submitted to the IEC for approval before implementation.

ii. **Participant Communication:**

CONFIDENTIAL

a. Inform participants of any intervention changes, the rationale, and potential impacts on safety and well-being.

b. Provide additional education and resources to address identified risks.

iii. **Emergency Protocols:**

a. Develop and communicate protocols for addressing severe adverse events or complications.

b. Ensure staff are trained in emergency response procedures to protect participant safety.

iv. **Final Evaluation and Reporting:**

a. Conduct a comprehensive analysis of risks and benefits observed throughout the study.

b. Prepare a report for the IEC and stakeholders detailing findings, including management of serious adverse events.

CONFIDENTIAL

3 OBJECTIVES AND ENDPOINTS

The primary healthcare intervention package (Multi-FrAME) is expected to improve health outcomes for older adults with multimorbidity and frailty, including a reduction in frailty progression and enhanced functional ability. This will assess the following outcomes.

**Objectives**	**Endpoints**	**Justification For Endpoints**	**Putative Mechanisms Of Action**
3.1 PRIMARY OBJECTIVES			
Assess the effectiveness of the Multi-FrAME package in improving health-related quality of life	Change in EQ-5D-5L index scores at 0, 6, and 12 months	EQ-5D-5L is validated for use in India and measures multiple dimensions of health	Comprehensive care improves physical, mental, and social health
3.2 SECONDARY OBJECTIVES			
Evaluate improvement in frailty	Change in frailty index scores (deficit accumulation model)	Reliable measure for frailty changes	Nutritional support and physical activity reduce frailty
Assess improvement in ADL/IADL	Change in ADL/IADL scores using Lawton IADL Scale	Validated measure for functional status	Improved physical and cognitive functioning
Decrease healthcare utilization	Reduction in healthcare visits and hospital admissions	Reflects stabilized health conditions	Coordinated care reduces the need for acute services
Decrease healthcare expenditure	Reduction in total healthcare costs	Economic impact of reduced healthcare spending	Efficient management techniques

Improve medication adherence	Increase in Medication Adherence Reporting Scale (MARS) scores	Indicates adherence behavior changes	Education and psychosocial support improve adherence
Increase patient satisfaction	Higher scores in Patient Satisfaction Questionnaires	Direct feedback on care experience	Patient-centered care enhances satisfaction
Reduce mortality rates	Reduction in all-cause mortality over study duration	Key determinant of long-term effectiveness	Comprehensive care strategies prolong life

CONFIDENTIAL

3.3 Biochemical and Haematological Outcomes:

i. **Blood Glucose Levels:**

a. **Outcome Measurement:** Change in glycosylated hemoglobin and random blood sugar levels.

b. **Justification:** Indicates metabolic health and risk for conditions like diabetes.

ii. **Lipid Profile:**

a. **Outcome Measurement:** Changes in LDL, HDL, Cholesterol and triglycerides.

b. **Justification:** Reflects cardiovascular health improvements.

iii. **Liver and Kidney Function Tests:**

a. **Outcome Measurement:** Changes in enzyme levels (e.g., ALT, AST) and creatinine (along with eGFR).

b. **Justification:** Indicates improvements in organ function.

iv. **Complete Blood Count (CBC):**

a. **Outcome Measurement:** Hemoglobin and hematocrit levels. Along with WBC ratios.

b. **Justification:** Indicates overall hematological health and potential for anemia improvement.

v. **Micronutrient Levels:**

a. **Outcome Measurement:** Changes in Vitamin D, iron, and calcium levels.

b. **Justification:** Provides insight into nutritional and bone health improvements.

vi. **Thyroid Function Tests:**

a. **Outcome Measurement:** Changes in TSH, T3, and T4 levels. Changes in testosterone, oestrogen and progesterone levels

b. **Justification:** Reflects endocrine health and potential metabolic improvements.

CONFIDENTIAL

4 STUDY DESIGN

4.1 OVERALL DESIGN

This is a phase 3 cluster randomized, parallel arm, observer-blinded trial. The clusters will be urban Primary Healthcare Centres (PHCs- but also known as Health and Wellness Centres or Ayushman

Arogya Mandirs) in Bhubaneswar city, randomly assigned to either the Multi-FrAME intervention or standard care.

4.2 Scientific Rationale for Study Design

The use of a cluster randomized design is essential in this trial to effectively evaluate the Multi-FrAME intervention's impact within the urban Primary Healthcare Centres (PHCs) of Bhubaneswar city. Given the nature of the intervention, which includes various components at the healthcare center level, randomizing at the cluster level reduces contamination risks between participants receiving the intervention and those receiving standard care.

This design is particularly relevant in the context of Bhubaneswar's urban PHCs, where healthcare practices can vary significantly. By employing a cluster approach, the trial can assess the intervention in a way that mirrors real-world application, enhancing external validity. Randomization at the PHC level allows for the capture of data reflecting the complex interactions and systemic changes induced by the intervention. This will also improve future possibility of uptake into systems as the intervention is being tested at the point of proposed delivery through the system.

The observer-blinded aspect of the study further strengthens its validity by reducing assessment bias. Blinding ensures that outcome evaluators remain unaware of which group (intervention or control) the participants belong to, thus minimizing potential biases in data collection and interpretation.

CONFIDENTIAL

4.3 Justification for Intervention

The Multi-FrAME package is designed to address the multifaceted challenges posed by multimorbidity and frailty among older adults, conditions prevalent in the urban population of Bhubaneswar. Traditional care models often fall short in managing these issues comprehensively.

The intervention includes integrated components: medical management, nutritional support, tailored physiotherapy, structured physical activity, and psychosocial counselling. These elements are combined to provide holistic care that aims to enhance health-related quality of life, reduce healthcare utilization, and improve overall health outcomes.

By implementing this intervention at the PHC level, the trial leverages existing healthcare infrastructure, promoting sustainability and scalability. The Multi-FrAME package is expected to equip healthcare providers with the necessary tools to deliver patient-centered care that is customized to meet the specific needs of older adults with multimorbidity and frailty, ultimately improving individual and population health outcomes.

The focus on holistic care is supported by evidence indicating that multidimensional interventions can lead to improved physical function, better management of chronic conditions, and enhanced mental well-being, thereby addressing gaps left by standard care practices.

4.4 END-OF-STUDY DEFINITION

The study will conclude after the 12-month follow-up data collection for all participants. This will include data analysis and preparation of findings for dissemination.

5 STUDY POPULATION:

5.1 INCLUSION CRITERIA

- Adults aged 60 years and above.
- Patients with multimorbidity, defined by two or more chronic health conditions, as assessed by the MAQ-PC plus tool(Pati et al., 2016).
- Frailty index score of ≥ 0.25 (Ghosh et al., 2023).
- Residents of Bhubaneswar city.

5.2 EXCLUSION CRITERIA

- Severe cognitive impairment or dementia as determined by the DART (Dementia Assessment Rapid Test)(B & V, 2015).
- Bedridden patients.

CONFIDENTIAL

- History of significant cardiac health problems- (disorders with specific requirement by clinicians to avoid Maximal and Sub-maximal physical activities)

- Grade III Osteoarthritis or other severe mobility impairments.

- Participants unable or unwilling to provide informed consent.

5.3 Lifestyle Considerations

Field investigators will assess lifestyle factors through comprehensive interviews and standardized questionnaires. Key areas to evaluate include:

- **Physical Activity Levels:** Use the International Physical Activity Questionnaire (IPAQ) to assess regular physical activities, including walking, household chores, and exercise routines.

- **Dietary Habits:** A 24-hour dietary recall will be conducted to understand nutritional intake. Investigators should focus on the consumption of fruits, vegetables, protein, calcium, and iron-rich foods.

- **Social Engagement:** Assess social interactions and support systems using instruments like the Lubben Social Network Scale. We will consider involvement in community activities and family support dynamics.

5.4 Screen Failures

Investigators will carefully document screen failures to ensure transparent reporting and allow for process improvements. Procedures include:

- **Initial Screening:** Conducted through interviews using the MAQ-PC plus tool and DART test.

- **Criteria Check:** Investigators will ensure each criterion is meticulously verified. Document reasons for exclusion, such as failing the frailty index threshold or positive dementia screening.

CONFIDENTIAL

- **Data Recording:** We will maintain a log of screen failures for audit purposes, including demographic details and reasons for exclusion, without including identifiable information in analyses.

5.5 Strategies for Recruitment and Retention

Recruitment:

- **Partnerships:** Collaborate with local health workers and community leaders who are trusted by potential participants. We will make attempts to recruit participants in equal proportions from each gender and age groups.

- **Awareness Programs:** Organize sessions in local community centers to explain the study's benefits and procedures.

- **Informed Consent:** We will provide clear, simple explanations and answer questions to ensure participants fully understand the consent form. This will be done using vernacular languages (English, Odia or Hindi) and visual aids.

CONFIDENTIAL

Retention:

- **Regular Follow-up:** We will schedule regular check-ins through phone calls or home visits to maintain participant engagement.

- **Feedback Mechanisms:** We will use a telephone hotline to create channels for participants to express concerns or difficulties, ensuring timely resolution.

- **Transportation Assistance:** We would offer support for participants facing challenges in traveling to PHCs.

- **Reward Systems:** We would provide small incentives to cover loss of wages as per ethical norms and certificates of appreciation to acknowledge participation.

6 STUDY INTERVENTION(S) OR EXPERIMENTAL MANIPULATION(S)

6.1 Study Intervention Overview

The Multi-FrAME package will be implemented at the cluster level across urban PHCs. It includes comprehensive components tailored for addressing multimorbidity and frailty:

- **Medical Management:** Regular review and optimization of medication regimens, as well as tools to improve medication adherence.
- **Nutritional Support:** Education on balanced diets and supplementation with Vitamin D3, iron, folic acid, calcium, and protein as needed.
- **Physiotherapy and Physical Activity:** Customized exercise programs focusing on resistance and aerobic activities.
- **Psychosocial Support:** Individual and group sessions to support mental health and enhance social connections. Health promotion materials provided for self-help.

6.2 ADMINISTRATION AND/OR DOSING

- **Intervention Delivery:** Conducted by trained healthcare professionals at participating PHCs, assisted by trained investigators from the study team.
- **Monthly Contact Sessions:** All testing, data collection, clinical management, physiotherapy, and physical activity reviews conducted during these sessions.
- **Health Diary:** Each participant will receive a "Health Diary," serving multiple purposes:
  - Intervention Log: Enables participants and caregivers to maintain a daily record, helping verify intervention fidelity.
  - Documentation Hub: A single point for filing lab reports and documenting other medications and health updates during the trial.
  - Information, Education, and Communication (IEC): Contains materials for health promotion, behavior change, and education. Participants can refer to these resources for ongoing learning and support.

CONFIDENTIAL

Intervention Category	Specifications	Dose/Frequency	Mode of Administration	Self-care/Caregiver Required
Medical Management	Medication review and optimization; Diary for improving adherence and compliance	Monthly, with diary data being updated daily	In-person at PHC	Self-care, with caregiver assistance if needed
Nutritional Support	Vitamin D3, iron, folic acid, calcium, and protein supplements	Doses are subject to the results of participants' biochemical tests for the respective nutrients. These doses will be monitored and modified as necessary to maintain nutrient levels within the biological reference range.	Oral supplementation	Self-care
Physiotherapy and Physical Activity	Customized exercise program	Monthly sessions, with daily exercises	In-person sessions and home-based exercises	Self-care, with caregiver support for safety
Psychosocial Support	Individual and group therapy sessions (Including therapeutic games, storytelling, friendship enrichment program), health promotion through the health diary IEC material	Breathing exercise and Meditation on daily basis, Psychological Counselling as per the patient's requirement, and Group sessions once in 2-3 month	In-person or via tele-health	Self-care

CONFIDENTIAL

Details of the intervention are provided as an additional file.

6.3 FIDELITY

Interventionist Training and Tracking: To ensure intervention fidelity, regular training, supervision, and monitoring will be implemented. The intervention will be delivered using the existing infrastructure and resources of the Health and Wellness Centres (HWCs). Study team facilitators will ensure adherence to protocols through:

- **Training:** Consistent and thorough training for all interventionists to maintain standard delivery practices.
- **Supervision:** Ongoing supervision by the study team to oversee intervention implementation.
- **Monitoring:** Routine monitoring to track adherence to the planned intervention protocol.

Daily Intervention and Health Diary:

- Each patient will receive a daily administered intervention and a health diary for recording their daily intake or practice of the intervention.
- The health diary will include detail doses, frequency, timing, and other relevant information.
- Participants are required to update the health diary daily.
- Investigators will collect and review health diary data monthly during contact sessions.

Follow-up Assessments:

- Comprehensive follow-up assessments will occur every six months at the health facility, where participants will undergo outcome evaluations.

6.4 MEASURES TO MINIMIZE BIAS

Randomization and Blinding

- **Randomization:** PHCs will be randomized to intervention or control groups to reduce selection bias.

CONFIDENTIAL

- **Observer Blinding:** Outcomes will be assessed by blinded evaluators to minimize measurement bias.

6.5 Study Intervention/Experimental Manipulation Adherence

- **Data Collection:** Monthly collections from the Health Diary during contact sessions to ensure adherence and fidelity.

- **Monthly Check-ins:** Provide additional support and address any barriers to compliance.

7 CONCOMITANT THERAPY

Participants are advised to continue any existing prescribed therapies unless contraindicated by the trial protocol.

8 DISCONTINUATION AND PARTICIPANT DISCONTINUATION/WITHDRAWAL

8.1 Discontinuation of Study Intervention/Experimental Manipulation:

Participants may discontinue the intervention under the following circumstances:

- **Adverse Events:** If a participant experiences an adverse event that poses a risk to their health or well-being, determined by the investigator, intervention may be paused or discontinued.

- **Non-compliance:** Repeated non-compliance with the intervention protocol may lead to discontinuation after consultative discussions by the study team.

- **Participant Request:** Participants can voluntarily withdraw from the intervention at any time without affecting their standard medical care.

- **Investigator's Decision:** The investigator may decide to discontinue the intervention if it's in the participant's best interest, based on emerging safety data or other concerns.

8.2 Participant Discontinuation/Withdrawal from the Study:

CONFIDENTIAL

Participants may withdraw from the study due to:

- **Voluntary Withdrawal:** Participants have the right to withdraw consent and discontinue participation at any stage. They will be encouraged to complete a final assessment for safety data.
- **Loss to Follow-up:** If participants miss three consecutive scheduled visits and cannot be contacted despite reasonable efforts, they will be considered lost to follow-up.
- **Discovery of Ineligibility:** If found ineligible during the study, participants will be withdrawn, and the reasons documented.

8.3 Procedures for Discontinuation/Withdrawal

- **Documentation:** All discontinuations or withdrawals will be documented, including the reason and date.
- **Data Management:** Data collected up to the point of withdrawal will be retained and used in analyses unless the participant requests otherwise.
- **Continuous Communication:** Study staff will maintain regular communication to address concerns and potentially prevent withdrawal.

The principles of respect, safety, and voluntary participation will guide all decisions related to discontinuation and withdrawal. Participants will receive appropriate counselling and support throughout this process.

9 STUDY ASSESSMENTS AND PROCEDURES

This section outlines the comprehensive approach for standardizing data collection, measurement modalities, and assessment procedures for the trial. The aim is to ensure consistency, reliability, and validity across all sites and participants.

9.1 Endpoint and Other Non-Safety Assessments

A. Primary Endpoint:

CONFIDENTIAL

- **Health-Related Quality of Life:**
    - **Tool:** EQ-5D-5L Index (EuroQol Group, 1990)
    - **Assessment:** Administered at 0, 6, and 12 months. Investigator administered.

B. Secondary Endpoints:

i. **Frailty Index:**

a. **Tool:** Cumulative Deficit Frailty Index (Rockwood et al., 2005)

b. **Details of the index:** The study will employ a deficit-based frailty index (FI) to assess frailty status, following the cumulative deficit model (Ghosh et al., 2023). The FI will be constructed using 40 health-related variables covering multiple domains, including chronic diseases, functional limitations, cognitive impairments, and self-perceived health status. Each variable will be coded as 0 (deficit absent), 1 (deficit present), or assigned an intermediate value (e.g., 0.5) for partial deficits. The FI score for each participant will be calculated by dividing the total number of deficits present by the total number of variables assessed, resulting in a score ranging from 0 to 1. Based on this score, participants will be categorized as robust (FI ≤0.15), prefrail (FI >0.15 to ≤0.24), or frail (FI >0.25). This method provides a comprehensive and standardized approach to measuring frailty by accounting for the accumulation of health deficits across multiple domains.

c. **Assessment Schedule:** Assessed at baseline, 6, and 12 months.

ii. **ADL/IADL:**

a. **Tool:** Lawton and Brody Instrumental Activities of Daily Living Scale (Lawton & Brody, 1969)

b. **Assessment Schedule:** Conducted at baseline, 6, and 12 months.

iii. **Healthcare Utilization:**

CONFIDENTIAL

a. **Tool:** Custom questionnaire capturing healthcare visits and hospital admissions.

b. **Data Collection:** Monthly via participant health diaries and healthcare records.

iv. **Healthcare Expenditure:**

a. **Assessment:** Monthly data collection from participant reports and receipt submissions.

v. **Medication Adherence:**

a. **Tool:** Medication Adherence Reporting Scale (MARS) (Thompson et al., 2000)

b. **Frequency:** Assessed at 0, 6, and 12 months.

vi. **Patient Satisfaction:**

a. **Tool:** Patient Satisfaction Questionnaire (Greenfield & Attkisson, 1989)

b. **Frequency:** Evaluated at 6, and 12 months.

vii. **Mortality Rates:**

a. **Data Collection:** Continuous monitoring throughout the study period using health records and family reports.

CONFIDENTIAL

9.2 SAFETY ASSESSMENTS

- **Adverse Events:**
  - **Definition:** Any undesirable experience associated with the use of the intervention.
  - **Assessment Frequency:** Continuous monitoring with mandatory reporting within 24 hours of occurrence.

- **Severity Classification:** Mild, Moderate, Severe based on impact on daily activities.

- **Serious Adverse Events:**
  - **Definition:** Events resulting in death, hospitalization, disability, or jeopardizing participant health.
  - **Reporting:** Immediate notification to Principal Investigator and ethics board, with documentation filed timely.

- **Vital Signs and Routine Checks:**
  - **Parameters:** Blood pressure, heart rate, weight, and BMI.
  - **Frequency:** Monthly during contact sessions.

- **Lab Tests:** (also as outcomes as described above in section 3.2)
  - **Assessment Schedule:** 0, 6, and 12 months.
  - **Tests Included:** (detailed as endpoints in section 3.2)
    - **Biochemical Metrics:** Blood glucose, lipid profile, liver function tests, kidney function tests.
    - **Hematological Metrics:** Complete blood count, hemoglobin levels.
    - **Micronutrient Profiles:** Levels of Vitamin D, iron, and calcium.
    - **Hormonal Profiles:** Thyroid function tests and other relevant hormone levels.

9.3 Data Collection and Management

- **Health Diaries:**
  - Participants will maintain daily logs.
  - Collected monthly for centralized data entry and analysis.

CONFIDENTIAL

- **Standard Operating Procedures (SOPs):**
  - Detailed SOPs will guide assessors in administering tools and conducting interviews to ensure consistency.
- **Training of Field Staff:**
  - All staff and assessors will undergo training sessions using detailed manuals, including mock assessments and role plays to ensure precision and reliability in data collection.
  - Refresher trainings will be held at 6 month interval and in case of staff turnover.
  - The Manual of Operating Procedures is prepared and will be used for training.

10 ADVERSE EVENTS AND SERIOUS ADVERSE EVENTS

10.1 Definition of Adverse Events (AEs)

An adverse event (AE) is any undesirable medical occurrence in a trial participant that may present during the trial, whether related to the intervention or not. This includes any new symptoms or worsening of pre-existing conditions.

10.2 Definition of Serious Adverse Events (SAEs)

A serious adverse event (SAE) is an AE that results in any of the following outcomes:

- Death
- Life-threatening condition
- Hospitalization or prolongation of existing hospitalization
- Persistent or significant disability/incapacity
- Any other medically important condition

10.3 Classification of an Adverse Event

CONFIDENTIAL

- **Severity of Event:**
    - Mild: Temporary and easily tolerated.
    - Moderate: Causes discomfort and interrupts normal activities.
    - Severe: Incapacitating and significantly impacts daily life.
- **Relationship to Study Intervention/Experimental Manipulation:**
    - Not related: Clearly due to other causes.
    - Unlikely related: Temporal relationship but unlikely to be associated.
    - Possibly related: Temporal relationship with a possible causal link.
    - Probably related: Likely caused by the intervention.
    - Definitely related: A direct result of the intervention.

10.4 Time Period and Frequency for Event Assessment and Follow-Up

- AEs will be assessed continuously throughout the trial.
- Participants will be followed up telephonically through a hotline number for the duration of the trial. This will be bi-directional, with participants having the ability to report AE anytime they face.
- Participants will be monitored monthly during contact sessions.
- Any AE reported will be followed up until resolution or stabilization.

10.5 Adverse Event Reporting

- AEs will be documented in the participant's case report form within 24 hours of awareness.

CONFIDENTIAL

- UDetails to include: description, onset date, severity, relationship to intervention, and outcome.
- Relationship with AE and intervention: This will be decided by a panel of clinicians and advisory board experts along with investigators to classify each AE as described above in 8.3.3.
- Regular Regular review (quarterly) to ensure timely reporting and management.

10.6 Serious Adverse Event Reporting

- SAEs will be reported immediately (within 24 hours) to the Principal Investigator and ethics committee, along with the managing clinician.
- A detailed SAE report will include all relevant medical information and follow-up actions.

10.7 Reporting Events to Participants

- Participants will be informed of any AEs or SAEs related to their participation.
- Communication will be clear and compassionate, with explanations of implications and any necessary actions.
- All other lab investigations and reports will be communicated to the participants through routine follow-up channels of the trial.

10.8 Events of Special Interest

- Events of special interest include those that might not meet SAE criteria but are significant due to the nature of the intervention (e.g., unexpected physiological changes).
- These will be monitored closely and reported similarly to SAEs to facilitate understanding of intervention-related risks.

CONFIDENTIAL

11 UNANTICIPATED PROBLEMS

11.1 DEFINITION OF UNANTICIPATED PROBLEMS

Unanticipated problems refer to incidents, experiences, or outcomes that are unexpected in nature, severity, or frequency given the research setting and relevant subject population. These problems may affect the safety, rights, or well-being of participants or others involved in the trial.

11.2 UNANTICIPATED PROBLEMS REPORTING

- **Immediate Reporting:** All unanticipated problems will be reported to the Principal Investigator and ethics committee within 24 hours of identification.
- **Documentation:** A thorough incident report will be created detailing the nature of the problem, circumstances of occurrence, and any immediate corrective actions taken.
- **Review and Action:** The ethics committee will periodically review the report to determine further actions, including protocol amendments or participant safety measures.

CONFIDENTIAL

11.3 REPORTING UNANTICIPATED PROBLEMS TO PARTICIPANTS

- **Timely Communication:** Participants affected by unanticipated problems will be informed promptly. The communication will provide a clear explanation of the problem, potential impacts, and any changes to their involvement or care.
- **Support and Guidance:** Participants will receive appropriate support and counselling to address concerns and ensure their continued well-being and informed participation.

12 STATISTICAL CONSIDERATIONS

12.1 STATISTICAL HYPOTHESES

- **Null Hypothesis (H0):** The Multi-FrAME intervention package has no effect on frailty progression, functional ability, health-related quality of life, healthcare utilization, or life satisfaction compared to routine care.

- **Alternative Hypothesis (HA):** The Multi-FrAME intervention package significantly improves frailty progression, functional ability, health-related quality of life, healthcare utilization, or life satisfaction compared to routine care.

12.2 Sample Size Determination

- **Assumptions:**
    - Mean Quality of life utility scores of control group = 0.87 [SD=0.17] (Jyani et al., 2023)
    - Effect size: Based on anticipated (clinically meaningful) improvement of 10% in HRQoL scores (Kshatri et al., 2025)
    - Power: 90%
    - Significance level: 0.05
    - Intra-cluster correlation: 0.24 (Kshatri et al., 2023)
    - Attrition rate: 25%
- **Calculation Methodology:** The following steps were used for the calculation of sample size

1. Effect size calculation

Effect size is the standardized mean difference between the control and intervention groups: calculated using the following formula,

$$Effect\ Size\ (Cohen's\ d) = \frac{Mean\ difference\ (Improvement)}{SD}$$

2. Sample size for two sample t-tests

The formula to calculate the sample size for a two-sample t-test is based on the following

$$n = \frac{(Z_{1-\propto/2} + Z_{1-\beta})^2}{d} * \frac{2(SD)^2}{Effect\ size}$$

Where:

CONFIDENTIAL

- $Z_{1-\alpha/2}$ is the critical value for a two-tailed test at the significance level αα.
- $Z_{1-\beta}$ is the critical value corresponding to the desired power (1−β).
- d is the effect size.
- SD is the standard deviation

The pwr.t.test() function in R is used to perform this calculation, which outputs the required sample size for each arm of the trial assuming a simple t-test.

3. Design Effect Adjustment for Clustering

In cluster randomized trials, the design effect is used to adjust for the fact that individuals within the same cluster are likely to be more similar to each other than to individuals in other clusters. The design effect (DE) is calculated as:

$$Design\ Effect\ (DE) = 1 + (m - 1) * ICC$$

$$n_{adjusted} = n * DE$$

Where:

- m is the number of participants per cluster.
- ICC is the intraclass correlation coefficient.

4. Adjustment for Attrition:

Finally, the sample size is adjusted for expected attrition (dropout) by dividing the adjusted sample size by the retention rate:

$$n_{final} = \frac{n_{adjusted}}{1 - Attrition\ Rate}$$

5. Sample Size per Cluster

The final sample size per cluster is calculated by dividing the adjusted and attrition-corrected sample size by the number of clusters:

CONFIDENTIAL

$$Sample\ size\ per\ cluster = \frac{n_{final}}{Number\ of\ clusters}$$

12.3 Populations for Analyses

The analysis will use the Intention-to-Treat (ITT) approach, which includes all randomized participants, regardless of their level of adherence to the intervention or any withdrawals from the study. This method preserves the benefits of randomization, allowing an assessment of the intervention's effectiveness under real-world conditions by accounting for typical variations in participant behaviour and engagement. By including all randomized individuals, the ITT analysis offers a realistic estimate of the intervention's impact on the general population represented in the study.

12.4 Statistical Analyses

12.4.1 General Approach:

The study will ensure secure data storage with controlled access to maintain participant confidentiality. Data management procedures will be implemented to ensure data accuracy, completeness, and integrity, with regular quality checks to address any discrepancies or missing data. The data analysts will be blinded to the groups. Descriptive analysis of the trial data will summarize participant characteristics, including demographics, baseline health status, and distribution of chronic conditions, with means, standard deviations, medians, and frequencies calculated as appropriate. Data will be analyzed (intention to treat) using appropriate statistical methods, such as mixed-effects models to account for clustering. We will compare the outcomes between the intervention and control groups to assess the effectiveness of the Multi-FrAME intervention, adjusting for clustering. Further, we will conduct subgroup analyses to explore potential moderators (e.g., age, gender) or mediators (e.g., adherence to the intervention) of the intervention effect.

CONFIDENTIAL

12.4.2 Analysis of the Primary Endpoint(s)

- **Outcome:** Health-related quality of life measured by EQ-5D-5L.
- **Method:** The primary outcome of this study is health-related quality of life, assessed using the EQ-5D-5L scale. The EQ-5D-5L instrument measures quality of life across five domains

(mobility, self-care, usual activities, pain/discomfort, and anxiety/depression), with each domain scored on five levels of severity. An overall utility score for quality of life will be calculated by mapping these responses to a single index value, facilitating comparison across participants. This index value is a continuous interval measure that allows for nuanced assessment of changes in health status. Primary outcome measures will be compared between the intervention and control groups using appropriate statistical tests based on the nature of the outcome variables (e.g., t-tests, chi-square tests, or non-parametric tests) to assess differences in outcomes. We will use adjusted Odds ratios arrived at using mixed effect models to calculate effect sizes and corresponding confidence intervals to quantify the magnitude of intervention effects.

12.4.3 Analysis of the Secondary Endpoint(s)

- **Outcomes:** Frailty progression, ADL/IADL scores, healthcare utilization, costs, patient satisfaction, medication adherence, mortality, along with all the laboratory measurements.

- **Method:** Each secondary outcome will be clearly defined and analyzed to evaluate the broader impacts of the intervention on participants' health and well-being. Frailty progression will be assessed through a frailty score as a continuous measure. Functional limitations will include work-limiting conditions, mobility, Activities of Daily Living (ADL), and Instrumental Activities of Daily Living (IADL), scored on an ordinal scale, using categorical measures (e.g., “able” vs. “unable”). Health care utilization will be measured by tracking healthcare events (e.g., visits, hospitalizations) in a binary or categorical format, while health care expenditure will be recorded as a continuous variable, capturing both mean and median expenditures. Medical adherence will be represented as a percentage, indicating the proportion of prescribed medications participants adhered to. Statistical comparisons of these secondary outcomes between the intervention and control groups will employ appropriate tests for each outcome variable type to assess differences. Mixed-effect models will calculate adjusted odds ratios to determine effect sizes, with corresponding confidence intervals to quantify the intervention's impact.

12.4.4 Safety Analyses:

CONFIDENTIAL

Frequency and severity comparisons across groups in terms of AE and SAE reported and other details from documentation in safety reports.

12.4.5 Baseline Descriptive Statistics:

- **Descriptive Analysis:** Summarize participant characteristics, baseline health status, and distribution of chronic conditions, and outcome measures including frailty and multimorbidity.

- **Comparative Analysis:** Analyze outcomes between intervention and control groups using appropriate statistical models (e.g., mixed-effects models).

12.4.6 Statistical Tools and Software:

Analyses conducted using statistical software R, with codes double checked, ensuring precise and reproducible results.

12.4.7 Planned Interim Analyses:

- **Objective:** To assess the safety and efficacy of the Multi-FrAME intervention at an intermediate point, ensuring ongoing participant safety and verifying preliminary effectiveness results.
- **Timing:** The interim analysis will be conducted after 50% of participants have completed the 12-month follow-up.
- **Criteria:**
  - **Safety:** Evaluate the incidence and severity of adverse and serious adverse events.
  - **Efficacy:** Assess primary and secondary outcomes, focusing on health-related quality of life (EQ-5D-5L) and frailty index changes.
- **Statistical Considerations:** The analysis will not impact the overall type I error rate as it's not stopping the trial for efficacy. We will use descriptive statistics to identify any emerging trends or safety concerns.
- **Decision-Making Process:** An independent data monitoring committee (DMC) will review interim results. Recommendations will be made regarding the continuation, modification, or early termination of the trial based on predefined criteria.

CONFIDENTIAL

- **Reporting:** Findings will be documented and shared with the ethics committee without full disclosure to the research team, unless necessary for participant safety.

12.4.8 Sub-Group Analyses

- **Objective:** To explore differential effects of the Multi-FrAME intervention among various participant subgroups, providing deeper insights into specific population benefits.
- **Subgroups of Interest:**
  - **Age Categories:**
    - <70 years
    - ≥70 years
  - **Gender:**
    - Male
    - Female
  - **Baseline Frailty Level:**
    - Mild frailty (Frailty Index < 0.30)
    - Moderate frailty (Frailty Index ≥ 0.30)
  - **Comorbidity Categories:**
    - 2-3 chronic conditions
    - >3 chronic conditions
  - **Adherence to interventions**- each component
  - **Sensitivity analysis by excluding contamination of control arm** – If any interventions are received/taken by t themselves in the control arm participants

CONFIDENTIAL

- **Analysis Methods:**
    - **Statistical Approach:** Use interaction terms in regression models to examine variations in treatment effects across subgroups.
    - **Outcome Measures:** Analyze primary and secondary outcomes within each subgroup, focusing on health-related quality of life, frailty index, and healthcare utilization.
- **Interpretation:**
    - Identify trends that may indicate which subgroups benefit most from the intervention.
    - Consider clinical significance alongside statistical significance to guide potential tailoring of interventions in future applications.
- **Reporting:**
    - Subgroup findings will be reported in terms of effect sizes and confidence intervals to highlight meaningful differences.
    - These analyses will be exploratory and not adjusted for multiple comparisons, interpreted cautiously.

CONFIDENTIAL

12.4.9 TABULATION OF INDIVIDUAL PARTICIPANT DATA

In this study, individual participant data will be systematically tabulated by measure and time point to facilitate comprehensive analysis and interpretation of the results. Each participant's data will be organized into a structured format, allowing for easy access and review of specific outcomes at designated follow-up intervals.

12.4.10 EXPLORATORY ANALYSES

- **Objective:** To investigate additional patterns and unexpected findings that emerge from the data, offering insights beyond the predefined hypotheses.

- **Scope:**
    - **Baseline Characteristics Influence:** Examine how various baseline characteristics (e.g., socioeconomic status, lifestyle factors) might influence outcomes.
    - **Longitudinal Changes:** Assess trends over time across different outcomes to capture subtle, ongoing effects of the intervention.
    - **Novel Biomarkers:** Explore the potential role of novel biomarkers or laboratory parameters that were not primary or secondary outcomes.

- **Approach:**
    - **Descriptive Statistics:** Use descriptive measures to identify potential correlations and patterns.
    - **Multivariate Analyses:** Apply multivariate models to explore complex interactions between variables.
    - **Data Visualization:** Employ advanced visualization techniques to uncover hidden relationships and trends.
- **Interpretation:**
    - Insights from exploratory analyses will help generate hypotheses for future research.
    - Findings will be interpreted with caution, acknowledging the exploratory nature and the lack of control for multiple comparisons.
- **Reporting:**
    - Results will be documented in supplementary materials or appendices, highlighting potential areas for further investigation.
    - Exploratory findings will guide researchers in refining interventions and informing larger-scale studies.

CONFIDENTIAL

13 SUPPORTING DOCUMENTATION AND OPERATIONAL CONSIDERATIONS

13.1 Regulatory, Ethical, and Study Oversight Considerations

We have obtained ethical approval from the Institutional Human Ethical Committee of ICMR-RMRC Bhubaneswar. We will adhere to the ICMR National Ethical Guidelines for biomedical research involving human participants throughout the trial.

13.2 Informed Consent Process

- **Procedure:** We will ensure that the informed consent process is comprehensive and understandable. Investigators will introduce participants to the study details, including its purpose, procedures, potential risks, and benefits.

- **Language and Comprehension:** Consent forms and Participants Information Sheets will be provided in the participant's preferred language, and verbal explanations will accompany written documents.

- **Assessment of Understanding:** Investigators will assess participants' understanding by asking them to describe the study in their own words and clarify any questions.

- **Documentation:** Signed consent forms will be securely stored, and participants will receive a copy along with a detailed participant information sheet.

13.3 Study Discontinuation and Closure

- **Responsibility:** The Principal Investigator (PI) will oversee the study closure process, collaborating with the trial coordinator and admin assistant.

- **Procedure:** Upon reaching the study endpoint or if early termination is necessary, we will conduct a final data review and ensure all interventions and data collections are complete.

- **Reporting:** A detailed closure report will be submitted to the ethics committee and stakeholders, summarizing the study outcomes and any issues encountered.

13.4 Confidentiality and Privacy

- **Data Protection Principles:** We will use secure validated electronic data capture (EDC) tools and a unified database management system (DBMS) to manage trial data, accessible only by the PI and statisticians.

- **Retrieval for Monitoring:** Authorized personnel will retrieve data for monitoring and interim analysis using secure systems, ensuring only de-identified data is shared for analysis.

CONFIDENTIAL

- **Findings Review:** The statistical team will analyze data, reviewed by the steering committee, and findings will be shared with the research team and ethics board.

13.5 Future Use of Stored Specimens and Data

- **Consent:** We will obtain consent for any future research use of stored data or specimens.
- **Sample Management:** Samples will be cataloged and securely stored following ethical agreements for potential future research.

13.6 Key Roles and Study Governance

- **Principal Investigator (PI):** Leads the study, ensuring compliance and overseeing all operations.
- **Co-Investigators and Collaborators:** Provide expertise from clinical and related backgrounds, supporting intervention delivery.
- **Trial Manager and Coordinator:** Handle daily operations, logistics, and coordination with research staff, organize monitoring and QA/QC measures.
- **Laboratory In-charge:** Oversees sample collection, transport, IPC practices, testing, and reporting.
- **Statistical Team:** Conducts data analysis and interim evaluations.
- **Admin Assistant and Research Staff:** Assist in data collection, documentation, and participant interactions.
- **Technicians and PHC Collaborators:** Conduct lab tests and support intervention delivery.
- **Steering Committee:** Provides strategic oversight and governance to ensure alignment with study objectives.
- **Organogram:**

CONFIDENTIAL

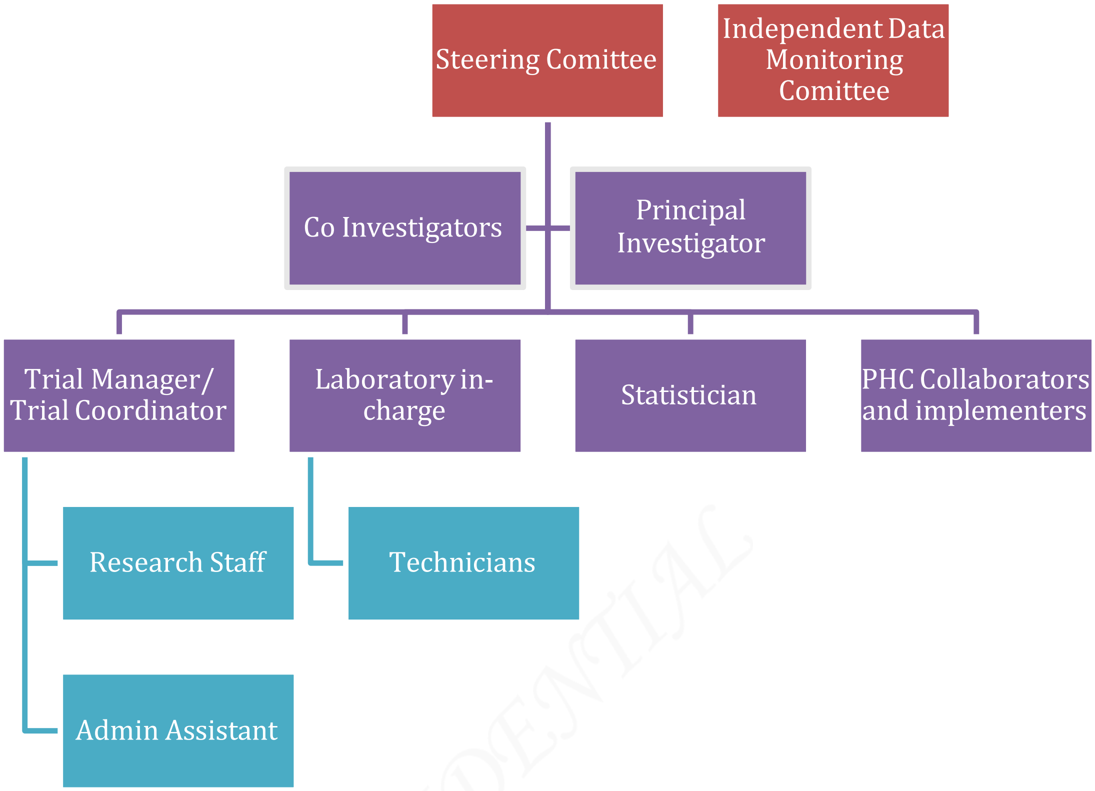

CONFIDENTIAL

13.7 Safety Oversight

- **Drug, Data and Safety Monitoring Board (DSMB):** We will utilize a DSMB to independently review safety data and advise on study conduct. The DSMB will meet after 33%, 66% and 100% completion of study timelines.

- **Safety Reviews:** We will conduct regular safety assessments to identify any emerging issues promptly. Any change or deviation in protocol will be conveyed to the DSMB immediately and taken up for review in the subsequent meeting.

13.8 Clinical Monitoring

- **Monitoring Plan:** We will implement routine monitoring of trial sites by both internal and external experts for verifying compliance with protocols and ensuring data integrity.
- **Site Visits:** Regular visits by independent monitors will facilitate adherence to protocols and address any emerging issues immediately.

13.9 Quality Assurance and Quality Control

- **Lab and Measurement QA/QC:**
    - **Sample Collection and Transport:** Samples will be collected and transported adhering to strict IPC practices to ensure integrity, as detailed in the MOPs.
    - **Testing and Reporting:** Our laboratory personnel will conduct tests, logging results into the trial database securely.

13.10 Data QA/QC:

- **Data Management System:** We will use advanced EDC tools for accurate data entry and unified DBMS for centralized data management. The tools are provided in the MOPs and annexures.
- **Audit Trails:** An audit trail will track all data handling activities to ensure transparency.

13.11 Data Handling and Record Keeping

- **Secure Systems:** We will store all data in secure, password-protected databases, with access restricted to authorized personnel.
- **Documentation:** Detailed records of all data handling activities will be maintained to ensure accuracy.
- All study data will be stored securely in source formats for a duration of 5 years from the completion of the trial, as per the ethical committee requirements.

13.12 Protocol Deviations:

CONFIDENTIAL

We will document any protocol deviations and assess their potential impact on the study integrity, implementing corrective actions as necessary.

13.13 Publication and Data Sharing Policy:

Authorship will be based on significant contributions, with all contributors duly acknowledged, following ICMJE criteria for authorship. We will share de-identified data in accordance with ethical guidelines and open-access policies where applicable.

13.14 Conflict of Interest Policy:

We will require all study personnel to disclose potential conflicts of interest, implementing strategies to mitigate these and protect study integrity

14 ADDITIONAL CONSIDERATIONS

14.1 Cultural Sensitivity:

- **Community Engagement:** We will actively involve community leaders and stakeholders to ensure the trial is culturally sensitive and aligns with local customs.
- **Participant Support:** Tailored communication strategies will be employed to address cultural and linguistic diversity, ensuring comprehension and engagement.

14.2 Participant Burden:

- **Minimizing Disruption:** Study procedures will be scheduled at convenient times to accommodate participants' daily routines, minimizing inconvenience.
- **Compensation:** Participants will receive appropriate compensation for their time and travel to participate in the study.

14.3 Contingency Planning:

- **Adverse Events:** Predefined protocols will guide the management of any unexpected events, ensuring prompt attention and care.

- **Resource Allocation:** Sufficient resources, including staff and materials, will be allocated to handle unforeseen challenges smoothly.

14.4 Environmental Considerations:

- **Sustainability:** Efforts will be made to minimize environmental impact by reducing waste and promoting the use of sustainable materials throughout the study.

- **Facility Readiness:** Trial sites will be evaluated for their preparedness and suitability to ensure a conducive environment for study activities.

14.5 Technology and Innovation:

- **Telehealth Integration:** Explore opportunities for using telehealth to reach participants who may face mobility challenges or geographic barriers.

- **Data Technology:** Leverage advanced data collection and management technologies to enhance accuracy and efficiency

15 ABBREVIATIONS AND SPECIAL TERMS

- **PHC**: Primary Healthcare Centre
- **EQ-5D-5L**: EuroQol 5-Dimensions, 5 Levels
- **MAQ-PC**: Multi-Condition Assessment Questionnaire for Primary Care
- **DART**: Dementia Assessment Rapid Test
- **ICMR**: Indian Council of Medical Research
- **RMRC**: Regional Medical Research Centre
- **HWC**: Health and Wellness Centre
- **EDC**: Electronic Data Capture

CONFIDENTIAL

- **DBMS**: Database Management System
- **PI**: Principal Investigator
- **DMC**: Data Monitoring Committee
- **SOPs**: Standard Operating Procedures
- **ICT**: Information and Communication Technology
- **QA/QC**: Quality Assurance/Quality Control
- **ICC**: Intraclass Correlation Coefficient
- **SAE**: Serious Adverse Event
- **AE**: Adverse Event
- **PROMs**: Patient-Reported Outcome Measures
- **ADL/IADL**: Activities of Daily Living/Instrumental Activities of Daily Living
- Protocol Amendment History- NA

CONFIDENTIAL

List of CRFS for the trial

CRF	**Purpose**	**Time Point**	**Key Constructs/Variables**
Eligibility CRF	Assess participant eligibility	Screening	Inclusion/exclusion criteria, consent status
Recruitment/Baseline CRF	Collect initial participant data	Baseline	Demographics, baseline health status, medical history
Follow-up CRF	Track ongoing participant status and outcomes	6, 12, months	Health-related quality of life, frailty index, ADL/IADL, medication adherence, healthcare utilization

Exit CRF	Record reasons for study exit	Upon exit	Mortality, migration, withdrawal of consent, ineligibility
Endline/Completion CRF	Capture final outcomes and status	End of study	Health status, final lab results, intervention outcomes
Telephone Log CRF	Document phone interactions	Continuous	Contact details, communication notes, follow-up reminders
Fidelity of Intervention CRF	Monitor intervention delivery and adherence	Continuous	Adherence to protocol, intervention delivery records
Monitoring CRF	Ensure protocol compliance and data integrity	Continuous	Monitoring findings, corrective actions
AE/SAE CRF	Document adverse and serious adverse events	As needed	Event description, severity, relation to intervention, outcome

CONFIDENTIAL

16 REFERENCES:

1. United Nations, Department of Economic and Social Affairs, Population Division. World Population Prospects 2022.
2. Barnett K, et al. Epidemiology of multimorbidity and implications for health care. Lancet. 2012;380(9836):37-43.
3. Fried LP, et al. Frailty in Older Adults: Evidence for a Phenotype. J Gerontol A Biol Sci Med Sci. 2001;56(3):M146-M156.
4. Collard RM, et al. Prevalence of frailty in community-dwelling older persons: a systematic review. J Am Geriatr Soc. 2012;60(8):1487-1492.
5. Chang SF, et al. Prevalence and factors associated with frailty in the community-dwelling older population. Nurs Res. 2019;68(3):276-287.
6. Dent E, et al. The association between frailty and quality of life: a systematic review. Age Ageing. 2016;45(3):456-462.
7. Clegg A, et al. Interventions for frailty: a systematic review. Ageing Ment Health. 2013;17(6):683-693.
8. Puts MT, et al. Interventions to prevent or reduce the level of frailty in community-dwelling older adults: A systematic review and meta-analysis. Ageing Res Rev. 2017;37:138-155.
9. Hoogendijk EO, et al. Frailty: implications for clinical practice and public health. Lancet. 2019;394(10206):1365-1375.
10. Pati, S., Hussain, M. A., Swain, S., Salisbury, C., Metsemakers, J. F. M., Knottnerus, J. A., & Van Den Akker, M. (2016). Development and Validation of a Questionnaire to Assess Multimorbidity in Primary Care: An Indian Experience. BioMed Research International, 2016(1), 6582487. https://doi.org/10.1155/2016/6582487
11. Ghosh, A., Kundu, M., Devasenapathy, N., Woodward, M., & Jha, V. (2023). Frailty among middle-aged and older women and men in India: findings from wave 1 of the longitudinal Ageing study in India. BMJ Open, 13(7), e071842. https://doi.org/10.1136/BMJOPEN-2023-071842
12. B, S., & V, S. (2015). Dementia Assessment by Rapid Test (DART): An Indian Screening T ool for Dementia. Journal of Alzheimer's Disease & Parkinsonism, 05(03). https://doi.org/10.4172/2161-0460.1000198
13. Maher RL, et al. Clinical consequences of polypharmacy in elderly. Expert Opin Drug Saf. 2014;13(1):57-65.
14. Gnjidic D, et al. Impact of high-risk drug use on hospitalization and mortality in older people. Drugs Aging. 2012;29(8):615-627.

CONFIDENTIAL

15. de Labra C, et al. Effects of physical exercise interventions in frail older adults: A systematic review of randomized controlled trials. BMC Geriatr. 2015;15:154.
16. Landi F, et al. Exercise as a remedy for sarcopenia. Curr Opin Clin Nutr Metab Care. 2018;21(1):25-31.
17. Bischoff-Ferrari HA, et al. A meta-analysis of vitamin D and calcium in the treatment of osteoporosis. Osteoporos Int. 2016;27(3):893-902.
18. Fairweather-Tait SJ, et al. Iron and folic acid supplementation in pregnancy and risk of maternal mortality and adverse birth outcomes. Cochrane Database Syst Rev. 2014;Issue 12:CD012342.
19. Bauer JM, et al. Nutritional treatment of frailty syndrome in older persons: A systematic review and meta-analysis. J Am Med Dir Assoc. 2013;14(9):611-622.
20. Wuthrich VM, et al. A randomized controlled trial of the Cool Kids/Cool Teens programs to prevent anxiety and depression in young people. Behav Res Ther. 2015;75:65-75.
21. Chodosh J, et al. Cognitive behavioral treatment for depression in frail older adults: A randomized controlled trial. JAMA. 2005;293(9):1063-1069.
22. Kshatri JS, et al. Prevalence and Patterns of Multimorbidity Among Rural Elderly: Findings of the AHSETS Study. Front Public Health. 2020;8:582663.
23. Kshatri JS, et al. Is multimorbidity associated with risk of elder abuse? Findings from the AHSETS study. BMC Geriatr. 2021;21:413.
24. Kshatri JS, et al. Associations of multimorbidity on frailty and dependence among an elderly rural population: Findings from the AHSETS study. Mech Ageing Dev. 2020;192:111384.
25. Kshatri JS, et al. Improving health outcomes among older adults in India: Effectiveness and implementability of a novel comprehensive geriatric assessment based intervention. Wellcome Open Res. 2023;8:414.
26. Jones G. Pharmacokinetics of vitamin D toxicity. Am J Clin Nutr. 2008;88(2):582S-586S.
27. Holick MF. Vitamin D deficiency. N Engl J Med. 2007;357(3):266-281.
28. Vieth R. Vitamin D supplementation, 25-hydroxyvitamin D concentrations, and safety. Am J Clin Nutr. 1999;69(5):842-856.
29. Curhan GC, et al. A prospective study of dietary calcium and other nutrients and the risk of symptomatic kidney stones. N Engl J Med. 1997;33(12):833-838.
30. Straub DA. Calcium supplementation in clinical practice: A review of forms, doses, and indications. Nutr Clin Pract. 2007;22(3):286-296.
31. Haas JD, Brownlie T IV. Iron deficiency and reduced work capacity: A critical review of the research to determine a causal relationship. J Nutr. 2001;131(2S-2):676S-688S.

CONFIDENTIAL

32. Borgna-Pignatti C, et al. Thalassemia. Lancet. 2010;376(9731):155-167.
33. Provan D. Mechanisms and management of iron deficiency anaemia. Br J Haematol. 1999;107(1):19-26.
34. Chanarin I, Metz J. Folic acid deficiency and cognitive impairment in the elderly. Br Med J. 1997;315(7115):91-92.
35. Tamura T, Picciano MF. Folate and human reproduction. Am J Clin Nutr. 2006;83(6):1207-1215.
36. Macera CA, et al. Injury prevention: Physical activity-related injuries: Recommendations and interventions. Am J Prev Med. 2003;25(3 Suppl 1):57-63.
37. Thompson PD, et al. Exercise and acute cardiovascular events: Placing the risks into perspective. Circulation. 2007;115(17):2358-2368.
38. O'Halloran PD, et al. Prospective study of cardiac rehabilitation in older adults. J Cardiopulm Rehabil Prev. 2007;27(5):312-319.
39. Baikie KA, Wilhelm K. Emotional and physical health benefits of expressive writing. Adv Psychiatr Treat. 2005;11(5):338-346.
40. Tong A, et al. Research priorities in CKD: Report of a national workshop conducted in Australia. Nephrology (Carlton). 2012;17(8):725-732.
41. Cumming RG. Intervention in aged care facilities to reduce depression. Age Ageing. 1996;25(5):362-366.
42. Geraci JM, et al. Nosocomial infections in geriatric patients. Clin Geriatr Med. 2009;25(2):355-372.
43. Clarke DM, et al. Somatic symptoms, hypochondriasis and mental disorder in various cultures. Psychol Med. 2013;43(6):1213-1221.
44. Adlins B, et al. Managing chronic illness: The benefits of coordinated care. J Chronic Dis. 2014;9(3):192-199.
45. EuroQol Group. EuroQol—a new facility for the measurement of health-related quality of life. Health Policy. 1990;16(3):199-208.
46. Rockwood K, et al. A Frailty Index based on common clinical data: The cumulative deficits model. J Gerontol A Biol Sci Med Sci. 2005;60(5):478-484.
47. Greenfield TK, Attkisson CC. A meta-analysis of the satisfaction with healthcare services. Eval Program Plann. 1989;12(1):41-63.
48. Jyani, G., Prinja, S., Garg, B., Kaur, M., Grover, S., Sharma, A., & Goyal, A. (2023). Health-related quality of life among Indian population: The EQ-5D population norms for India. Journal of Global Health, 13. https://doi.org/10.7189/JOGH.13.04018

CONFIDENTIAL

49. Kshatri, J. S., AK, K., Rehman, T., Bhattacharya, H., Bhuyan, D., Mansingh, A., Sahoo, U. K., Nayak, M., Kanungo, S., Bhattacharya, D., & Pati, S. (2025). A comprehensive assessment of health indicators among tribal populations in Odisha, India (Odisha Tribal Family Health Survey): a community-based, cross-sectional study. The Lancet Regional Health - Southeast Asia, 38, 100611. https://doi.org/10.1016/J.LANSEA.2025.100611
50. Kshatri, J. S., Mansingh, A., Kavitha, A. K., Bhattacharya, H., Bhuyan, D., Bhattacharya, D., Rehman, T., Swain, A., Mishra, D., Tripathy, I., Mohapatra, M. R., Nayak, M., Sahoo, U. K., & Pati, S. (2023). Odisha tribal family health survey: methods, tools, and protocols for a comprehensive health assessment survey. Frontiers in Public Health, 11, 1157241. https://doi.org/10.3389/FPUBH.2023.1157241/BIBTEX

17 ANNEXURES

1. **Details of Interventions**
2. **Health Diary**
3. **CRFs and data collection forms**
4. **Consent forms**
5. **Project Brief**
6. **Laboratory report forms**
7. **AE/SAE reporting forms**
8. **Manual of operating procedures**
9. **ToR of DSMB members**

CONFIDENTIAL
