# Supplementary material for: Effectiveness and implementation of a primary healthcare intervention for multimorbidity and frailty among urban older adults in India: Protocol for the Multi-FrAME cluster randomized trial
Source: PLoS One. 2026 Jun 18;21(6):e0351110. doi: 10.1371/journal.pone.0351110 (PMC13278581; doi:10.1371/journal.pone.0351110)
Supplement: Attachment — Submitted filename: Response to Reviewers.pdf [file pone.0351110.s005.pdf]

Response to Reviewers

Title: Effectiveness and Implementation of a Primary Healthcare Intervention for Multimorbidity and Frailty Among Urban Older Adults in India: Protocol for the Multi-FrAME Cluster Randomized Trial

Manuscript Id: PONE-D-25-57404R1

Reviewer's comments

Sl No	Reviewer's Comments	Author's Response
1	Thank you for the opportunity to review this revised manuscript. The manuscript has improved, and my previous concerns and identified shortcomings have been adequately addressed. I have no further comments.	Thank you for your positive assessment of the study. We are thankful for the opportunity to further strengthen the protocol based on your suggestions.
2	Thank you for the edits and pentagon.<br><br>There is a section which mentions the statistical analysis is in the protocol. Please add the tests to the primary and secondary outcomes, and which difference between treatment values will be considered, and how these results will be interpreted with regard for conclusions.	Thank you for the query. The primary and secondary outcome of the manuscript has been revised.<br><br>In primary outcome the change in health-related quality of life (HRQoL) will be measured using the EQ-5D-5L index at baseline, 6 months, and 12 months.<br><br>The EQ-5D-5L index score will be analyzed as a continuous variable. Mean differences between intervention and control groups at 6 and 12 months, as well as changes from baseline, will be assessed using independent *t*-tests (or Mann–Whitney U tests if the data are non-normally distributed). To account for repeated measurements and intra-individual correlations over time, a linear mixed-effects model will be used, adjusting for baseline values and potential confounders.<br><br>Treatment effects will be expressed as adjusted mean differences between groups with 95% confidence intervals (CIs) and corresponding *p*-values. A statistically significant improvement in EQ-5D-5L scores in the intervention group compared to the control group will be interpreted as evidence of the effectiveness of the multicomponent intervention in improving HRQoL over time.

		Secondary outcomes will be analyzed according to their data type: continuous variables will be assessed using t-tests and linear mixed-effects models, categorical variables using chi-square tests and mixed-effects logistic regression, and ordinal outcomes using ordinal logistic models where appropriate. Treatment effects will be reported as adjusted mean differences or odds ratios with 95% confidence intervals, comparing intervention and control groups over time while adjusting for baseline differences.
3	The authors were able to address most of my comments. I have some additional minor recommendations to make: • The word "also" does not need to be said twice in the following sentence: "Also people in bedridden or mobility-limiting conditions such as Grade III osteoarthritis, having contraindications to physical activity, and unable or unwilling to participate in follow-up assessments will also be excluded from the study"	Thank you for your careful review and helpful suggestion. We agree that the repetition of the word "also" was unnecessary. The sentence has been revised under the heading "Exclusion Criteria"accordingly to improve clarity and readability.
	• The word "mediattion" should be "meditation" under the Psychosocial Support Heading.	Thank you for pointing out this typographical error. We have corrected "**mediattion**" to "**meditation**" under the Psychosocial Support heading.
	• Replace with "Participants who will be included" in the following sentence: "Participants who will include in control arm receive the routine care provided through the UPHC."	Thank you for this helpful suggestion. We have revised the sentence accordingly to "Participants in the control arm will receive routine care provided through the UPHC" under the heading "Control arm: routine standard care".
	• The word "whereas" should be replaced with "wherein" in this sentence: "The enrolment, interventions, and assessments schedule is presented in (S1_Figure) whereas a study flow diagram with study setting, screening and enrolment, cluster randomization, follow up assessment followed by mixed-effects regression analysis and dissemination is shown in (S2_Figure)"	Thank you for your helpful suggestion. We have modified the sentence as "The schedule of enrolment, interventions, and assessments is presented in **Figure 1 (The numbering of figures has been revised in the manuscript)**. A study flow diagram illustrating the study setting, screening and enrolment, cluster randomization, follow up assessment followed by mixed-effects regression analysis and dissemination is shown in **Figure 2 (The numbering of figures has been**

		**revised in the manuscript)** under the heading "Participant enrolment process and analysis plan".
	• The article still has a substantial amount of in-text errors. I strongly suggest the authors or editor(s) review and correct as appropriate before publication.	Thank you for this important observation. We have carefully reviewed the manuscript to identify and correct in-text errors and improve overall clarity, grammar, and consistency. The revised version has been thoroughly edited to ensure accuracy and readability prior to submission. The revised and edited text portions have been marked as yellow in the "Revised manuscript with track changes".

Editors comments

**Sl No**	**Editor's Comments**	**Author's Response**
**1**	If the reviewer comments include a recommendation to cite specific previously published works, please review and evaluate these publications to determine whether they are relevant and should be cited. There is no requirement to cite these works unless the editor has indicated otherwise.	Thank you for this suggestion. We carefully reviewed the reviewer comments and evaluated the relevance of the recommended publications. As no specific references were explicitly suggested for inclusion, no additional citations were incorporated into the revised manuscript.
2	Please review your reference list to ensure that it is complete and correct. If you have cited papers that have been retracted, please include the rationale for doing so in the manuscript text, or remove these references and replace them with relevant current references. Any changes to the reference list should be mentioned in the rebuttal letter that accompanies your revised manuscript. If you need to cite a retracted article, indicate the article's retracted status in the References list and also include a citation and full reference for the retraction notice.	Thank you for this important observation. We carefully reviewed the reference list to ensure that all citations are complete, accurate, and current. No retracted articles have been cited in the revised manuscript. Additionally, two references (**Reference 24 and Reference 28**) were updated and replaced with more relevant current references in the revised manuscript, and these changes have been highlighted using track changes.
