# Supplementary material for: Effectiveness and implementation of a primary healthcare intervention for multimorbidity and frailty among urban older adults in India: Protocol for the Multi-FrAME cluster randomized trial
Source: PLoS One. 2026 Jun 18;21(6):e0351110. doi: 10.1371/journal.pone.0351110 (PMC13278581; doi:10.1371/journal.pone.0351110)
Supplement: Attachment — Submitted filename: Editor Comments and Authors Response_v.3.docx [file pone.0351110.s003.docx]

**Response to Editor’s Comments**

| **Sl No** | **Editor’s Comments** | **Author’s Response** |
| --- | --- | --- |
| 1 | Please ensure that you include a title page within your main document. You should list all authors and all affiliations as per our author instructions and clearly indicate the corresponding author. | Title page listing all authors and all affiliations including corresponding author has been included within the manuscript and re submitted. |
