# Supplementary material for: Effectiveness and implementation of a primary healthcare intervention for multimorbidity and frailty among urban older adults in India: Protocol for the Multi-FrAME cluster randomized trial
Source: PLoS One. 2026 Jun 18;21(6):e0351110. doi: 10.1371/journal.pone.0351110 (PMC13278581; doi:10.1371/journal.pone.0351110)
Supplement: Attachment — Submitted filename: Response to Reviewer.docx [file pone.0351110.s004.docx]

**Response to Editor**

**Title: Effectiveness and Implementation of a Primary Healthcare Intervention for Multimorbidity and Frailty Among Urban Older Adults in India: Protocol for the Multi-FrAME Cluster Randomized Trial**

**Manuscript Id: PONE-D-25-57404**

**Editor’s comments**

| **Sl No** | **Editor’s Comments** | **Author’s Response** |
| --- | --- | --- |
| **1** | Thank you for stating the following financial disclosure:  [Indian Council of Medical Research (ICMR) Intramural Project Fund].  At this time, please address the following queries:  a) Please clarify the sources of funding (financial or material support) for your study. List the grants or organizations that supported your study, including funding received from your institution. | The study received financial support from Indian Council of Medical Research, New Delhi through Intramural funding scheme.  The study did not receive any institutional funding. |
|  | b) State what role the funders took in the study. If the funders had no role in your study, please state: “The funders had no role in study design, data collection and analysis, decision to publish, or preparation of the manuscript.” | “The funders had no role in study design, data collection and analysis, decision to publish, or preparation of the manuscript.”  The same has been mentioned in manuscript under the heading “Funding”. |
|  | c) If any authors received a salary from any of your funders, please state which authors and which funders. | NA |
|  | d) If you did not receive any funding for this study, please state: “The authors received no specific funding for this work.”  Please include your amended statements within your cover letter; we will change the online submission form on your behalf. | NA  We have included the amended statements within cover letter. |
| **2** | "It appears that your ORCiD iD has not been validated in your Editorial Manager account and we are unable to proceed until that step is complete.  To validate your ORCiD iD in Editorial Manager, please follow the steps below:  **A.** In your Editorial Manager account, please go to ‘Update my Information’ (in the upper left-hand corner of the main menu), and click on the Fetch/Validate link next to the ORCiD field. | As communicated to the PLOS editor in email dated on 2^nd^ February, 2026 stating that **“**I am writing to bring to your attention that we have an active submission in PLOS ONE (ref-PONE-D-25-57404). We have submitted this using my official email: [kshatri.js@icmr.gov.in](mailto:kshatri.js@icmr.gov.in). However, the system also requires me to linkmy ORCID account. Unfortunately my ORCID is linked to my other email: [jsk.icmr@outlook.com](mailto:jsk.icmr@outlook.com).  Due to this we are haveing trouble accessing the portal and competing the submission/revision.  I request you to kindly help with this challenge at the earliest.”  The authors received an reply from the editor on 4^th^ February stating that  “Dear Jaya Kshatri,  You had multiple user accounts in Editorial Manager for PLOS One. These records have now been combined into one record, including any authored submissions or current and past assignments. Your username for the remaining account is below.  Username: Jaya Kshatri  If you do not remember your password, please select the "Send Username/Password" link on the login page.  You can access Editorial Manager at <https://www.editorialmanager.com/pone/>. Once you have logged in, please review your personal information for accuracy by clicking on the "Update My Information" link on the menu.”  Unfortunately after combining the account we are still unable to fetch the ORCID Id nor we can link the existing ORCID ID. |
|  | **B.** This link will take you to the ORCiD site and allow you to create a new iD or authenticate a pre-existing iD in Editorial Manager.  For additional instructions, please watch the following video for a step-by-step demonstration: <https://www.youtube.com/watch?v=_xcclfuvtxQ>" | I would like to request to the editorial team if they can validate the existing ORCID ID with the registered email from their end as we are unable to do it.  **ORCID id: 0000-0003-3616-3402** |
| **3** | Can you please upload an additional copy of your revised manuscript that does not contain any tracked changes or highlighting as your main article file. This will be used in the production process if your manuscript is accepted. Please amend the file type for the file showing your changes to Revised Manuscript w/tracked changes. Please follow this link for more information: [http://blogs.PLOS.org/everyone/2011/05/10/how-to-submit-your-revised-manuscript/](http://blogs.plos.org/everyone/2011/05/10/how-to-submit-your-revised-manuscript/). | Thank you for your suggestion we have already submitted the cleaned manuscript which does not contain any track changes. |
| **4** | Your ethics statement should only appear in the Methods section of your manuscript. If your ethics statement is written in any section besides the Methods, please move it to the Methods section and delete it from any other section. Please ensure that your ethics statement is included in your manuscript, as the ethics statement entered into the online submission form will not be published alongside your manuscript. | It is mentioned in the methods section of the manuscript. Also updated in the submission portal. |
| **5** | We note that there were multiple versions of Manuscript, Response to Reviewers in your submission's file inventory. We have removed the older file(s), retaining the most recent version(s) for editorial review. Please double check your submission file inventory and let us know if any files appear to be outdated or absent. | We apologise for the inconvenience occurred. We have already removed the older version of files and updated with the new one. |
| **6** | We note that your author list was updated during the revision process. In order to add or remove authors or update the order of the author byline after initial submission, we ask that authors complete an Authorship Change Request form. You may review our full authorship change policy and download the Authorship Change Request form here: <https://journals.plos.org/plosone/s/authorship#loc-authorship-changes>.  Please fill out all required sections of the form. If you are adding or removing more than 2 authors, you can complete multiple forms and submit as many forms as needed to reflect the updates. Please return the Weform(s) as an attachment by emailing [plosone@plos.org](mailto:plosone@plos.org) or by uploading it as a submission file labeled with the file type ‘Other’. The form will be reviewed by PLOS staff for approval. Should the changes be approved, we will require written confirmation from all authors confirming that they approve of the changes. Please note that if your manuscript is accepted, we will not be able to complete the publication process without the completed form. | We have filled and completed the Authorship Change Request Form and uploaded as a submission file labelled with the file type ‘Other’. |
| **7** | PLOS follows the Committee on Publication Ethics (COPE: [https://publicationethics.org](https://publicationethics.org/)) guidelines for changes to authorship and therefore requires specific information relating to these changes. We would be grateful if you could, therefore, please provide us with the following:  ""Written confirmation from all authors - including the authors being removed/added - stating that they agree to the changes being made""  This written confirmation can be in the form of direct emails from individual authors to us, a compilation of emails provided by you, or a written statement signed by all co-authors.  As corresponding author, please could you contact all authors - including the authors being removed/added - to obtain this written confirmation. This written confirmation can be in the form of direct emails from individual authors to us, a compilation of emails provided by you, or a written statement signed by all co-authors. | A written statement signed by all co authors stating that “they agree to the changes being made” has been uploaded as Authors Declaration Form for the addition of new author Dr.Abhinav Sinha. |
