# Supplementary material for: Effectiveness and implementation of a primary healthcare intervention for multimorbidity and frailty among urban older adults in India: Protocol for the Multi-FrAME cluster randomized trial
Source: PLoS One. 2026 Jun 18;21(6):e0351110. doi: 10.1371/journal.pone.0351110 (PMC13278581; doi:10.1371/journal.pone.0351110)
Supplement: Peer review history

# Author comment: Author response to Decision Letter 0

**DOI:** 10.1371/journal.pone.0351110.r001

30 Oct 2025

Attachment Submitted filename: Editor Comments and Authors Response_v.3.docx

# Peer review: Decision Letter 0

**DOI:** 10.1371/journal.pone.0351110.r002

**Authors:** Ramesh Athe

11 Dec 2025

PONE-D-25-57404

Effectiveness and Implementation of a Primary Healthcare Intervention for Multimorbidity and Frailty Among Urban Older Adults in India: Protocol for the Multi-FrAME Cluster Randomized Trial

PLOS One

Dear Dr. Kshatri,

Thank you for submitting your manuscript to PLOS ONE. After careful consideration, we feel that it has merit but does not fully meet PLOS ONE’s publication criteria as it currently stands. Therefore, we invite you to submit a revised version of the manuscript that addresses the points raised during the review process.

Please submit your revised manuscript by Jan 25 2026 11:59PM. If you will need more time than this to complete your revisions, please reply to this message or contact the journal office at plosone@plos.org. When you're ready to submit your revision, log on to https://www.editorialmanager.com/pone/ and select the 'Submissions Needing Revision' folder to locate your manuscript file.

Please include the following items when submitting your revised manuscript:

- A rebuttal letter that responds to each point raised by the academic editor and reviewer(s). You should upload this letter as a separate file labeled 'Response to Reviewers'.
- A marked-up copy of your manuscript that highlights changes made to the original version. You should upload this as a separate file labeled 'Revised Manuscript with Track Changes'.
- An unmarked version of your revised paper without tracked changes. You should upload this as a separate file labeled 'Manuscript'.

If you would like to make changes to your financial disclosure, please include your updated statement in your cover letter. Guidelines for resubmitting your figure files are available below the reviewer comments at the end of this letter.

If applicable, we recommend that you deposit your laboratory protocols in protocols.io to enhance the reproducibility of your results. Protocols.io assigns your protocol its own identifier (DOI) so that it can be cited independently in the future. For instructions see: https://journals.plos.org/plosone/s/submission-guidelines#loc-laboratory-protocols. Additionally, PLOS ONE offers an option for publishing peer-reviewed Lab Protocol articles, which describe protocols hosted on protocols.io. Read more information on sharing protocols at https://plos.org/protocols?utm_medium=editorial-email&utm_source=authorletters&utm_campaign=protocols.

We look forward to receiving your revised manuscript.

Kind regards,

Ramesh Athe, PhD

Academic Editor

PLOS One

Journal Requirements:

1. Please ensure that your manuscript meets PLOS ONE's style requirements, including those for file naming. The PLOS ONE style templates can be found at

https://journals.plos.org/plosone/s/file?id=wjVg/PLOSOne_formatting_sample_main_body.pdf and

https://journals.plos.org/plosone/s/file?id=ba62/PLOSOne_formatting_sample_title_authors_affiliations.pdf

2. Thank you for stating the following financial disclosure:

[Indian Council of Medical Research (ICMR) Intramural Project Fund].

At this time, please address the following queries:

a) Please clarify the sources of funding (financial or material support) for your study. List the grants or organizations that supported your study, including funding received from your institution.

b) State what role the funders took in the study. If the funders had no role in your study, please state: “The funders had no role in study design, data collection and analysis, decision to publish, or preparation of the manuscript.”

c) If any authors received a salary from any of your funders, please state which authors and which funders.

d) If you did not receive any funding for this study, please state: “The authors received no specific funding for this work.”

Please include your amended statements within your cover letter; we will change the online submission form on your behalf.

3. When completing the data availability statement of the submission form, you indicated that you will make your data available on acceptance. We strongly recommend all authors decide on a data sharing plan before acceptance, as the process can be lengthy and hold up publication timelines. Please note that, though access restrictions are acceptable now, your entire data will need to be made freely accessible if your manuscript is accepted for publication. This policy applies to all data except where public deposition would breach compliance with the protocol approved by your research ethics board. If you are unable to adhere to our open data policy, please kindly revise your statement to explain your reasoning and we will seek the editor's input on an exemption. Please be assured that, once you have provided your new statement, the assessment of your exemption will not hold up the peer review process.

4. PLOS requires an ORCID iD for the corresponding author in Editorial Manager on papers submitted after December 6th, 2016. Please ensure that you have an ORCID iD and that it is validated in Editorial Manager. To do this, go to ‘Update my Information’ (in the upper left-hand corner of the main menu), and click on the Fetch/Validate link next to the ORCID field. This will take you to the ORCID site and allow you to create a new iD or authenticate a pre-existing iD in Editorial Manager.

5. Your ethics statement should only appear in the Methods section of your manuscript. If your ethics statement is written in any section besides the Methods, please move it to the Methods section and delete it from any other section. Please ensure that your ethics statement is included in your manuscript, as the ethics statement entered into the online submission form will not be published alongside your manuscript.

6. If the reviewer comments include a recommendation to cite specific previously published works, please review and evaluate these publications to determine whether they are relevant and should be cited. There is no requirement to cite these works unless the editor has indicated otherwise.

Additional Editor Comments (if provided):

[Note: HTML markup is below. Please do not edit.]

Reviewers' comments:

Reviewer's Responses to Questions

**Comments to the Author**

1. Does the manuscript provide a valid rationale for the proposed study, with clearly identified and justified research questions?

The research question outlined is expected to address a valid academic problem or topic and contribute to the base of knowledge in the field.

Reviewer #1: Yes

Reviewer #2: Yes

Reviewer #3: Yes

Reviewer #4: Yes

**********

2. Is the protocol technically sound and planned in a manner that will lead to a meaningful outcome and allow testing the stated hypotheses?

The manuscript should describe the methods in sufficient detail to prevent undisclosed flexibility in the experimental procedure or analysis pipeline, including sufficient outcome-neutral conditions (e.g. necessary controls, absence of floor or ceiling effects) to test the proposed hypotheses and a statistical power analysis where applicable. As there may be aspects of the methodology and analysis which can only be refined once the work is undertaken, authors should outline potential assumptions and explicitly describe what aspects of the proposed analyses, if any, are exploratory.

Reviewer #1: Partly

Reviewer #2: Yes

Reviewer #3: Yes

Reviewer #4: Yes

**********

3. Is the methodology feasible and described in sufficient detail to allow the work to be replicable?

Descriptions of methods and materials in the protocol should be reported in sufficient detail for another researcher to reproduce all experiments and analyses. The protocol should describe the appropriate controls, sample size calculations, and replication needed to ensure that the data are robust and reproducible.

Reviewer #1: Yes

Reviewer #2: No

Reviewer #3: Yes

Reviewer #4: Yes

**********

4. Have the authors described where all data underlying the findings will be made available when the study is complete?

The PLOS Data policy requires authors to make all data underlying the findings described in their manuscript fully available without restriction, with rare exception, at the time of publication. The data should be provided as part of the manuscript or its supporting information, or deposited to a public repository. For example, in addition to summary statistics, the data points behind means, medians and variance measures should be available. If there are restrictions on publicly sharing data—e.g. participant privacy or use of data from a third party—those must be specified.

Reviewer #1: Yes

Reviewer #2: No

Reviewer #3: Yes

Reviewer #4: Yes

**********

5. Is the manuscript presented in an intelligible fashion and written in standard English?

PLOS ONE does not copyedit accepted manuscripts, so the language in submitted articles must be clear, correct, and unambiguous. Any typographical or grammatical errors should be corrected at revision, so please note any specific errors here.

Reviewer #1: Yes

Reviewer #2: Yes

Reviewer #3: Yes

Reviewer #4: Yes

**********

6. Review Comments to the Author

Please use the space provided to explain your answers to the questions above and, if applicable, provide comments about issues authors must address before this protocol can be accepted for publication. You may also include additional comments for the author, including concerns about research or publication ethics.

You may also provide optional suggestions and comments to authors that they might find helpful in planning their study.

(Please upload your review as an attachment if it exceeds 20,000 characters)

Reviewer #1: Thank you for the opportunity to review this well written study protocol. This protocol describes a comprehensive study and includes many components, which overall are well detailed in the protocol and its appendices and addresses an important topic. However, there are a few points I would like to highlight in relation to potential improvements.

It would be beneficial if the different components of the intervention were clarified also in text (not only supplementary files), making it difficult to understand what will actually take place and what you believe will lead to positive outcomes for frail older adults in India compared to usual care (control group)

The measurements and questionnaires included in the study and the forthcoming analysis should be described in more detail in text (not only supplementary files), such as the type of questionnaires used (self-report or interview), the nature of the questions, etc. This is important for assessing validity and reliability, as well as for understanding what the study is actually evaluating.

Please clarify how the enrollment process will be performed.

The result of your power calculation should be included in the text.

Please include explanations of abbreviations in the figure captions.

Reviewer #2: What was the sample size determined in the calculation where individual patients would be randomised? What is the cluster size assumed to be? What is the final sample size intended to be? What if this is not obtained by November 30? Include the numbers in the body of the text.

In S figure 2 - is the frailty value intended to be 0.25 (not 0,25)?

Evaluation is spelled incorrectly.

In Sfigure 1 - why are there boxes after the 12 month visit? In the study the data collection stops with the 12 month QoL.

Will the QoL measures be scored according to any scoring manuals?

How will significance be assessed, or results interpreted?

Reviewer #3: The paper describes a promising intervention tailored to improving geriatric outcomes in older adults with multimorbidity. At this time, the paper has a noticeable amount of spelling and grammatical errors and thus some proofreading and editing of the entire manuscript is strongly suggested. There are also some key information that are needed to provide further context in the manuscript, highlighted as follows:

How will patients be recruited?

There are several components to the intervention – clinical, physical activity, medication etc. The authors should specify where these components will be disbursed concurrently on in an ordered manner (one after the other). How long will each of these components take? For example, are the exercises on a weekly basis? For medication management, how do you account for medications with a short time frame of administration?

The authors should comment on the feasibility of adding this intervention to routine clinical flow. If indeed the intervention is shown to improve outcomes, what tests will be conducted to show that it can be implemented and sustained on a wider scale in this region and potentially in the nation? Any anticipated challenges or limitations?

Reviewer #4: General Assessment

This manuscript presents a well-conceptualized and methodologically robust study protocol addressing an important public health challenge, the management of multimorbidity and frailty among older adults in urban India. The topic is highly relevant given the demographic transition, the rising burden of chronic conditions, and the need for evidence-based, scalable interventions in LMICs. The protocol is well aligned with current global priorities on healthy ageing and integrated primary healthcare.

Introduction

The introduction effectively outlines the global and regional burden of multimorbidity and frailty, clearly establishing the rationale for a multicomponent primary care intervention in LMIC settings. The research gap and justification are well-articulated, and the study objectives are logically framed to address these evidence gaps. The background successfully links the problem to the proposed intervention and highlights the need for system-level integration within urban primary health centres.

Methods

The methodological framework is detailed, transparent, and consistent with SPIRIT and CONSORT guidelines for cluster-randomised trials.

Key components—including trial design, study setting, eligibility criteria, intervention and control arms, medical management, nutritional support, physiotherapy, psychological support, and study outcomes, are described comprehensively. The inclusion of participant flow diagrams, sample size calculation, analysis plan, and data management procedures enhances the protocol’s reproducibility. Ethical considerations, data monitoring, and dissemination plans are clearly articulated and appropriately referenced.

The methodology demonstrates strong implementation feasibility within existing UPHC infrastructure, reflecting thoughtful integration with national health systems and community-based resources.

Discussion

The discussion section effectively summarises the novelty and potential public health implications of the trial. The authors clearly articulate how the study could inform the development of scalable, evidence-based primary care models for frailty and multimorbidity in resource-constrained settings. The acknowledgement of limitations and contextual challenges is balanced and realistic.

This trial has the potential to generate valuable insights into the effectiveness, feasibility, and scalability of multicomponent interventions for older adults in LMICs, thereby contributing to the global discourse on integrated care for ageing populations.

Overall Comment

This is a well-written and comprehensive protocol that demonstrates methodological rigor and public health significance. It addresses a critical gap in the management of frailty and multimorbidity through a contextually relevant, health system–integrated approach. The study’s findings are likely to have important implications for policy, primary healthcare strengthening, and geriatric service delivery in India and other LMICs.

**********

7. PLOS authors have the option to publish the peer review history of their article (what does this mean?). If published, this will include your full peer review and any attached files.

If you choose “no”, your identity will remain anonymous but your review may still be made public.

**Do you want your identity to be public for this peer review?**    For information about this choice, including consent withdrawal, please see our Privacy Policy.

Reviewer #1: No

Reviewer #2: No

Reviewer #3: No

Reviewer #4: **Yes:**   Jogesh Murmu

**********

[NOTE: If reviewer comments were submitted as an attachment file, they will be attached to this email and accessible via the submission site. Please log into your account, locate the manuscript record, and check for the action link "View Attachments". If this link does not appear, there are no attachment files.]

To ensure your figures meet our technical requirements, please review our figure guidelines: https://journals.plos.org/plosone/s/figures

You may also use PLOS’s free figure tool, NAAS, to help you prepare publication quality figures: https://journals.plos.org/plosone/s/figures#loc-tools-for-figure-preparation.

NAAS will assess whether your figures meet our technical requirements by comparing each figure against our figure specifications.

# Author comment: Author response to Decision Letter 1

**DOI:** 10.1371/journal.pone.0351110.r003

10 Apr 2026

Response to Reviewers has been submitted in the portal.

Attachment Submitted filename: Response to Reviewer.docx

# Peer review: Decision Letter 1

**DOI:** 10.1371/journal.pone.0351110.r004

**Authors:** Ramesh Athe, Sreeram Ramagopalan

27 Apr 2026

PONE-D-25-57404R1

Effectiveness and Implementation of a Primary Healthcare Intervention for Multimorbidity and Frailty Among Urban Older Adults in India: Protocol for the Multi-FrAME Cluster Randomized Trial

PLOS One

Dear Dr. Kshatri,

Thank you for submitting your manuscript to PLOS ONE. After careful consideration, we feel that it has merit but does not fully meet PLOS ONE’s publication criteria as it currently stands. Therefore, we invite you to submit a revised version of the manuscript that addresses the points raised during the review process.

Please submit your revised manuscript by Jun 11 2026 11:59PM. If you will need more time than this to complete your revisions, please reply to this message or contact the journal office at plosone@plos.org. When you're ready to submit your revision, log on to https://www.editorialmanager.com/pone/ and select the 'Submissions Needing Revision' folder to locate your manuscript file.

Please include the following items when submitting your revised manuscript:

- A letter that responds to each point raised by the academic editor and reviewer(s). You should upload this letter as a separate file labeled 'Response to Reviewers'.
- A marked-up copy of your manuscript that highlights changes made to the original version. You should upload this as a separate file labeled 'Revised Manuscript with Track Changes'.
- An unmarked version of your revised paper without tracked changes. You should upload this as a separate file labeled 'Manuscript'.

If you would like to make changes to your financial disclosure, please include your updated statement in your cover letter. Guidelines for resubmitting your figure files are available below the reviewer comments at the end of this letter.

If applicable, we recommend that you deposit your laboratory protocols in protocols.io to enhance the reproducibility of your results. Protocols.io assigns your protocol its own identifier (DOI) so that it can be cited independently in the future. For instructions see: https://journals.plos.org/plosone/s/submission-guidelines#loc-laboratory-protocols. Additionally, PLOS ONE offers an option for publishing peer-reviewed Lab Protocol articles, which describe protocols hosted on protocols.io. Read more information on sharing protocols at https://plos.org/protocols?utm_medium=editorial-email&utm_source=authorletters&utm_campaign=protocols.

As the corresponding author, your ORCID iD is verified in the submission system and will appear in the published article. PLOS supports the use of ORCID, and we encourage all coauthors to register for an ORCID iD and use it as well. Please encourage your coauthors to verify their ORCID iD within the submission system before final acceptance, as unverified ORCID iDs will not appear in the published article. *Only*    the individual author can complete the verification step; PLOS staff *cannot*    verify ORCID iDs on behalf of authors.

We look forward to receiving your revised manuscript.

Kind regards,

Sreeram V. Ramagopalan

Academic Editor

PLOS One

Journal Requirements:

1. If the reviewer comments include a recommendation to cite specific previously published works, please review and evaluate these publications to determine whether they are relevant and should be cited. There is no requirement to cite these works unless the editor has indicated otherwise.

2. Please review your reference list to ensure that it is complete and correct. If you have cited papers that have been retracted, please include the rationale for doing so in the manuscript text, or remove these references and replace them with relevant current references. Any changes to the reference list should be mentioned in the rebuttal letter that accompanies your revised manuscript. If you need to cite a retracted article, indicate the article’s retracted status in the References list and also include a citation and full reference for the retraction notice.

[Note: HTML markup is below. Please do not edit.]

Reviewers' comments:

Reviewer's Responses to Questions

**Comments to the Author**

1. Does the manuscript provide a valid rationale for the proposed study, with clearly identified and justified research questions?

The research question outlined is expected to address a valid academic problem or topic and contribute to the base of knowledge in the field.

Reviewer #1: Yes

Reviewer #2: Yes

Reviewer #3: Yes

**********

2. Is the protocol technically sound and planned in a manner that will lead to a meaningful outcome and allow testing the stated hypotheses?

The manuscript should describe the methods in sufficient detail to prevent undisclosed flexibility in the experimental procedure or analysis pipeline, including sufficient outcome-neutral conditions (e.g. necessary controls, absence of floor or ceiling effects) to test the proposed hypotheses and a statistical power analysis where applicable. As there may be aspects of the methodology and analysis which can only be refined once the work is undertaken, authors should outline potential assumptions and explicitly describe what aspects of the proposed analyses, if any, are exploratory.

Reviewer #1: Yes

Reviewer #2: Yes

Reviewer #3: Yes

**********

3. Is the methodology feasible and described in sufficient detail to allow the work to be replicable?

Descriptions of methods and materials in the protocol should be reported in sufficient detail for another researcher to reproduce all experiments and analyses. The protocol should describe the appropriate controls, sample size calculations, and replication needed to ensure that the data are robust and reproducible.

Reviewer #1: Yes

Reviewer #2: No

Reviewer #3: Yes

**********

4. Have the authors described where all data underlying the findings will be made available when the study is complete?

The PLOS Data policy requires authors to make all data underlying the findings described in their manuscript fully available without restriction, with rare exception, at the time of publication. The data should be provided as part of the manuscript or its supporting information, or deposited to a public repository. For example, in addition to summary statistics, the data points behind means, medians and variance measures should be available. If there are restrictions on publicly sharing data—e.g. participant privacy or use of data from a third party—those must be specified.

Reviewer #1: Yes

Reviewer #2: No

Reviewer #3: Yes

**********

5. Is the manuscript presented in an intelligible fashion and written in standard English?

PLOS ONE does not copyedit accepted manuscripts, so the language in submitted articles must be clear, correct, and unambiguous. Any typographical or grammatical errors should be corrected at revision, so please note any specific errors here.

Reviewer #1: Yes

Reviewer #2: Yes

Reviewer #3: Yes

**********

6. Review Comments to the Author

Please use the space provided to explain your answers to the questions above and, if applicable, provide comments about issues authors must address before this protocol can be accepted for publication. You may also include additional comments for the author, including concerns about research or publication ethics.

You may also provide optional suggestions and comments to authors that they might find helpful in planning their study.

(Please upload your review as an attachment if it exceeds 20,000 characters)

Reviewer #1: Thank you for the opportunity to review this revised manuscript. The manuscript has improved, and my previous concerns and identified shortcomings have been adequately addressed. I have no further comments.

Reviewer #2: Thank you for the edits and pentagon.

There is a section which mentions the statistical analysis is in the protocol. Please add the tests to the primary and secondary outcomes, and which difference between treatment values will be considered, and how these results will be interpreted with regard for conclusions.

Reviewer #3: The authors were able to address most of my comments. I have some additional minor recommendations to make:

• The word “also” does not need to be said twice in the following sentence: “Also people in bedridden or mobility-limiting conditions such as Grade III osteoarthritis, having contraindications to physical activity, and unable or unwilling to participate in follow-up assessments will also be excluded from the study”

• The word “mediattion” should be “meditation” under the Psychosocial Support Heading.

• Replace with “Participants who will be included” in the following sentence: “Participants who will include in control arm receive the routine care provided through the UPHC.”

• The word “whereas” should be replaced with “wherein” in this sentence: “The enrolment, interventions, and assessments schedule is presented in (S1_Figure) whereas a study flow diagram with study setting, screening and enrolment, cluster randomization, follow up assessment followed by mixed-effects regression analysis and dissemination is shown in (S2_Figure)”

• The article still has a substantial amount of in-text errors. I strongly suggest the authors or editor(s) review and correct as appropriate before publication.

**********

7. PLOS authors have the option to publish the peer review history of their article (what does this mean?). If published, this will include your full peer review and any attached files.

If you choose “no”, your identity will remain anonymous but your review may still be made public.

**Do you want your identity to be public for this peer review?**    For information about this choice, including consent withdrawal, please see our Privacy Policy.

Reviewer #1: No

Reviewer #2: No

Reviewer #3: No

**********

[NOTE: If reviewer comments were submitted as an attachment file, they will be attached to this email and accessible via the submission site. Please log into your account, locate the manuscript record, and check for the action link "View Attachments". If this link does not appear, there are no attachment files.]

To ensure your figures meet our technical requirements, please review our figure guidelines: https://journals.plos.org/plosone/s/figures

You may also use PLOS’s free figure tool, NAAS, to help you prepare publication quality figures: https://journals.plos.org/plosone/s/figures#loc-tools-for-figure-preparation.

NAAS will assess whether your figures meet our technical requirements by comparing each figure against our figure specifications.

# Author comment: Author response to Decision Letter 2

**DOI:** 10.1371/journal.pone.0351110.r005

8 May 2026

Reviewer’s comments 1

Thank you for the opportunity to review this revised manuscript. The manuscript has improved, and my previous concerns and identified shortcomings have been adequately addressed. I have no further comments.

Author’s Response 1

Thank you for your positive assessment of the study. We are thankful for the opportunity to further strengthen the protocol based on your suggestions.

Reviewer’s comments 2

Thank you for the edits and pentagon.

There is a section which mentions the statistical analysis is in the protocol. Please add the tests to the primary and secondary outcomes, and which difference between treatment values will be considered, and how these results will be interpreted with regard for conclusions.

Author’s Response 2

Thank you for the query. The primary and secondary outcome of the manuscript has been revised.

In primary outcome the change in health-related quality of life (HRQoL) will be measured using the EQ-5D-5L index at baseline, 6 months, and 12 months.

The EQ-5D-5L index score will be analyzed as a continuous variable. Mean differences between intervention and control groups at 6 and 12 months, as well as changes from baseline, will be assessed using independent t-tests (or Mann–Whitney U tests if the data are non-normally distributed). To account for repeated measurements and intra-individual correlations over time, a linear mixed-effects model will be used, adjusting for baseline values and potential confounders.

Treatment effects will be expressed as adjusted mean differences between groups with 95% confidence intervals (CIs) and corresponding p-values. A statistically significant improvement in EQ-5D-5L scores in the intervention group compared to the control group will be interpreted as evidence of the effectiveness of the multicomponent intervention in improving HRQoL over time.

Secondary outcomes will be analyzed according to their data type: continuous variables will be assessed using t-tests and linear mixed-effects models, categorical variables using chi-square tests and mixed-effects logistic regression, and ordinal outcomes using ordinal logistic models where appropriate. Treatment effects will be reported as adjusted mean differences or odds ratios with 95% confidence intervals, comparing intervention and control groups over time while adjusting for baseline differences.

Reviewer’s comments 3

The authors were able to address most of my comments. I have some additional minor recommendations to make:

• The word “also” does not need to be said twice in the following sentence: “Also people in bedridden or mobility-limiting conditions such as Grade III osteoarthritis, having contraindications to physical activity, and unable or unwilling to participate in follow-up assessments will also be excluded from the study”.

Author's Response 3

Thank you for your careful review and helpful suggestion. We agree that the repetition of the word “also” was unnecessary. The sentence has been revised under the heading “Exclusion Criteria”accordingly to improve clarity and readability.

Reviewer’s comments 3

• The word “mediattion” should be “meditation” under the Psychosocial Support Heading.

Author's Response 3

Thank you for pointing out this typographical error. We have corrected “mediattion” to “meditation” under the Psychosocial Support heading.

Reviewer’s comments 3

• Replace with “Participants who will be included” in the following sentence: “Participants who will include in control arm receive the routine care provided through the UPHC.”

Author's Response 3

Thank you for this helpful suggestion. We have revised the sentence accordingly to “Participants in the control arm will receive routine care provided through the UPHC” under the heading “Control arm: routine standard care”.

Reviewer’s comments 3

• The word “whereas” should be replaced with “wherein” in this sentence: “The enrolment, interventions, and assessments schedule is presented in (S1_Figure) whereas a study flow diagram with study setting, screening and enrolment, cluster randomization, follow up assessment followed by mixed-effects regression analysis and dissemination is shown in (S2_Figure)”.

Author's Response 3

Thank you for your helpful suggestion. We have modified the sentence as “The schedule of enrolment, interventions, and assessments is presented in Figure 1 (The numbering of figures has been revised in the manuscript). A study flow diagram illustrating the study setting, screening and enrolment, cluster randomization, follow up assessment followed by mixed-effects regression analysis and dissemination is shown in Figure 2 (The numbering of figures has been revised in the manuscript) under the heading “Participant enrolment process and analysis plan”.

Reviewer’s comments 3

• The article still has a substantial amount of in-text errors. I strongly suggest the authors or editor(s) review and correct as appropriate before publication.

Author's response

Thank you for this important observation. We have carefully reviewed the manuscript to identify and correct in-text errors and improve overall clarity, grammar, and consistency. The revised version has been thoroughly edited to ensure accuracy and readability prior to submission. The revised and edited text portions have been marked as yellow in the “Revised manuscript with track changes”.

Editors comments 1

If the reviewer comments include a recommendation to cite specific previously published works, please review and evaluate these publications to determine whether they are relevant and should be cited. There is no requirement to cite these works unless the editor has indicated otherwise.

Author's Response

Thank you for this suggestion. We carefully reviewed the reviewer comments and evaluated the relevance of the recommended publications. As no specific references were explicitly suggested for inclusion, no additional citations were incorporated into the revised manuscript.

Editors comments 2

Please review your reference list to ensure that it is complete and correct. If you have cited papers that have been retracted, please include the rationale for doing so in the manuscript text, or remove these references and replace them with relevant current references. Any changes to the reference list should be mentioned in the rebuttal letter that accompanies your revised manuscript. If you need to cite a retracted article, indicate the article’s retracted status in the References list and also include a citation and full reference for the retraction notice.

Author's Response

Thank you for this important observation. We carefully reviewed the reference list to ensure that all citations are complete, accurate, and current. No retracted articles have been cited in the revised manuscript. Additionally, two references (Reference 24 and Reference 28) were updated and replaced with more relevant current references in the revised manuscript, and these changes have been highlighted using track changes.

Attachment Submitted filename: Response to Reviewers.pdf

# Peer review: Decision Letter 2

**DOI:** 10.1371/journal.pone.0351110.r006

**Authors:** Ramesh Athe, Sreeram Ramagopalan, Sreeram Ramagopalan

24 May 2026

Effectiveness and Implementation of a Primary Healthcare Intervention for Multimorbidity and Frailty Among Urban Older Adults in India: Protocol for the Multi-FrAME Cluster Randomized Trial

PONE-D-25-57404R2

Dear Dr. Kshatri,

We’re pleased to inform you that your manuscript has been judged scientifically suitable for publication and will be formally accepted for publication once it meets all outstanding technical requirements.

Within one week, you’ll receive an e-mail detailing the required amendments. When these have been addressed, you’ll receive a formal acceptance letter and your manuscript will be scheduled for publication.

An invoice will be generated when your article is formally accepted. Please note, if your institution has a publishing partnership with PLOS and your article meets the relevant criteria, all or part of your publication costs will be covered. Please make sure your user information is up-to-date by logging into Editorial Manager at Editorial Manager® and clicking the ‘Update My Information' link at the top of the page. For questions related to billing, please contact billing support.

If your institution or institutions have a press office, please notify them about your upcoming paper to help maximize its impact. If they’ll be preparing press materials, please inform our press team as soon as possible -- no later than 48 hours after receiving the formal acceptance. Your manuscript will remain under strict press embargo until 2 pm Eastern Time on the date of publication. For more information, please contact onepress@plos.org.

Kind regards,

Sreeram V. Ramagopalan

Academic Editor

PLOS One

Additional Editor Comments (optional):

Reviewers' comments:

Reviewer's Responses to Questions

**Comments to the Author**

1. Does the manuscript provide a valid rationale for the proposed study, with clearly identified and justified research questions?

The research question outlined is expected to address a valid academic problem or topic and contribute to the base of knowledge in the field.

Reviewer #2: Yes

**********

2. Is the protocol technically sound and planned in a manner that will lead to a meaningful outcome and allow testing the stated hypotheses?

The manuscript should describe the methods in sufficient detail to prevent undisclosed flexibility in the experimental procedure or analysis pipeline, including sufficient outcome-neutral conditions (e.g. necessary controls, absence of floor or ceiling effects) to test the proposed hypotheses and a statistical power analysis where applicable. As there may be aspects of the methodology and analysis which can only be refined once the work is undertaken, authors should outline potential assumptions and explicitly describe what aspects of the proposed analyses, if any, are exploratory.

Reviewer #2: Yes

**********

3. Is the methodology feasible and described in sufficient detail to allow the work to be replicable?

Descriptions of methods and materials in the protocol should be reported in sufficient detail for another researcher to reproduce all experiments and analyses. The protocol should describe the appropriate controls, sample size calculations, and replication needed to ensure that the data are robust and reproducible.

Reviewer #2: Yes

**********

4. Have the authors described where all data underlying the findings will be made available when the study is complete?

The PLOS Data policy requires authors to make all data underlying the findings described in their manuscript fully available without restriction, with rare exception, at the time of publication. The data should be provided as part of the manuscript or its supporting information, or deposited to a public repository. For example, in addition to summary statistics, the data points behind means, medians and variance measures should be available. If there are restrictions on publicly sharing data—e.g. participant privacy or use of data from a third party—those must be specified.

Reviewer #2: Yes

**********

5. Is the manuscript presented in an intelligible fashion and written in standard English?

PLOS ONE does not copyedit accepted manuscripts, so the language in submitted articles must be clear, correct, and unambiguous. Any typographical or grammatical errors should be corrected at revision, so please note any specific errors here.

Reviewer #2: Yes

**********

6. Review Comments to the Author

Please use the space provided to explain your answers to the questions above and, if applicable, provide comments about issues authors must address before this protocol can be accepted for publication. You may also include additional comments for the author, including concerns about research or publication ethics.

You may also provide optional suggestions and comments to authors that they might find helpful in planning their study.

(Please upload your review as an attachment if it exceeds 20,000 characters)

Reviewer #2: Thank you for these edits, the statistical methods are clearly stated now, so it will be interesting to see the results in due course.

**********

7. PLOS authors have the option to publish the peer review history of their article (what does this mean?). If published, this will include your full peer review and any attached files.

If you choose “no”, your identity will remain anonymous but your review may still be made public.

**Do you want your identity to be public for this peer review?**    For information about this choice, including consent withdrawal, please see our Privacy Policy.

Reviewer #2: No

**********

# Editor report: Acceptance letter

**DOI:** 10.1371/journal.pone.0351110.r007

**Authors:** Ramesh Athe, Sreeram Ramagopalan, Sreeram Ramagopalan

PONE-D-25-57404R2

PLOS One

Dear Dr. Kshatri,

I'm pleased to inform you that your manuscript has been deemed suitable for publication in PLOS One. Congratulations! Your manuscript is now being handed over to our production team.

At this stage, our production department will prepare your paper for publication. This includes ensuring the following:

* All references, tables, and figures are properly cited

* All relevant supporting information is included in the manuscript submission,

* There are no issues that prevent the paper from being properly typeset

You will receive further instructions from the production team, including instructions on how to review your proof when it is ready. Please keep in mind that we are working through a large volume of accepted articles, so please give us a few days to review your paper and let you know the next and final steps.

Lastly, if your institution or institutions have a press office, please let them know about your upcoming paper now to help maximize its impact. If they'll be preparing press materials, please inform our press team within the next 48 hours. Your manuscript will remain under strict press embargo until 2 pm Eastern Time on the date of publication. For more information, please contact onepress@plos.org.

You will receive an invoice from PLOS for your publication fee after your manuscript has reached the completed accept phase. If you receive an email requesting payment before acceptance or for any other service, this may be a phishing scheme. Learn how to identify phishing emails and protect your accounts at https://explore.plos.org/phishing.

If we can help with anything else, please email us at customercare@plos.org.

Thank you for submitting your work to PLOS ONE and supporting open access.

Kind regards,

PLOS ONE Editorial Office Staff

on behalf of

Dr. Sreeram V. Ramagopalan

Academic Editor

PLOS One
